# Randomized Controlled Trials on Complementary and Traditional Medicine in the Korean Literature

**DOI:** 10.1155/2014/194047

**Published:** 2014-12-24

**Authors:** Chang-Kyu Kim, Da-Hee Kim, Myeong Soo Lee, Jong-In Kim, L. Susan Wieland, Byung-Cheul Shin

**Affiliations:** ^1^Department of Korean Medicine, School of Korean Medicine, Pusan National University, Yangsan 626-870, Republic of Korea; ^2^Medical Research Division, Korea Institute of Oriental Medicine, Daejeon 305-811, Republic of Korea; ^3^Department of Acupuncture & Moxibustion, Korean Medicine Hospital, Kyung Hee University, Seoul 130-872, Republic of Korea; ^4^Center for Integrative Medicine, University of Maryland School of Medicine, Baltimore, MD 21201, USA; ^5^Department of Rehabilitation Medicine, School of Korean Medicine, Pusan National University, Yangsan 626-870, Republic of Korea

## Abstract

*Objective*. This study aimed to identify all of the features of complementary and alternative (CAM) randomized controlled trials (RCTs) in the Korean literature and then introduce English-speaking researchers to the bibliometric and risk of bias characteristics of this literature.* Methods*. Eleven electronic databases and sixteen Korean journals were searched to August 2013 for RCTs of CAM therapies. Key study characteristics were extracted and risk of bias was assessed using the Cochrane Collaboration's tool for assessing risk of bias.* Results*. Three hundred and sixty publications met our inclusion criteria. Complementary and traditional medicine RCTs in the Korean literature emerged in the mid-1990s and increased in the mid-2000s. The most common CAM interventions include acupuncture (59.4%) and herbal medicine (8.3%). The largest proportion of trials evaluated CAM for musculoskeletal conditions (20.7%). Adequate methods of randomization were reported in 41.7% of the RCTs, whereas only 8.3% reported adequate allocation concealment. A low proportion of trials reported participant blinding (34.2%) and outcome assessor blinding (22.5%).* Conclusions*. Korean CAM RCTs are typically omitted from systematic reviews resulting in the potential for language bias. This study will enable these trials of diverse quality to be identified and assessed for inclusion in future systematic reviews on CAM interventions.

## 1. Introduction

Complementary and alternative medicine (CAM), as defined by National Center for Complementary and Alternative Medicine (NCCAM), is a group of diverse medical and health care systems, practices, and products that are not presently considered to be part of conventional medicine [1]. NCCAM currently classifies most CAM therapies into two broad categories: (1) natural products and (2) mind-body practices. However, some approaches may not neatly fit into either of these groups, for example, the practices of traditional Korean medicine (TKM) and traditional Chinese medicine (TCM) [2].

CAM has recently been increasingly popularized and introduced in Western societies, thus increasing the demand for research on its efficacy, including both controlled trials and systematic reviews. The complete identification of all potentially eligible controlled trials is a fundamental challenge in the preparation of systematic reviews [3]. Moreover it can be particularly challenging for systematic reviews of CAM interventions, because it is difficult to conduct a complete literature search that covers all relevant papers to avoid language, publication, and other possible biases [4]. Currently, many clinical trials and reviews on CAM are listed in English-based medical literature databases such as MEDLINE, EMBASE, and The Cochrane Library. The Cochrane CAM Field has devoted substantial efforts during the past decade to developing and maintaining a comprehensive register of controlled trials in CAM, which are contributed to The Cochrane Library Central Register of Controlled Trials (Central) [5].

Each Asian country has developed different ways of addressing traditional medicine modalities within its healthcare system. Korea has a unique dual healthcare delivery system that incorporates both traditional Korean and Western medicine [6]. For this reason, Korean trials of CAM interventions have usually been published in traditional medicine journals rather than Western CAM or conventional medicine journals, and Korean CAM trials are often not included in English-based core medical databases. However, searching Korean databases is challenging because many of Korea's medical literature citation databases cannot support English-language searches and there is no unified database. Because it is difficult to identify Korean CAM RCTs for inclusion in English-language systematic reviews, these trials have been omitted from some previous systematic reviews of acupuncture [7, 8]. This language barrier increases the risk of language bias [9]. The first step in addressing this gap in access to and use of the literature is to identify and describe current Korean RCTs in CAM. Though there are published descriptions of Korean trials in acupuncture [10], herbal medicine [11], tuina technique [12], and qi-gong therapy [13], most of these articles were published in Korean. To our knowledge, an English-language description of all known Korean RCTs in CAM has never been published.

For this reason, we aimed to update our previous work on Korean RCTs of acupuncture [10] and to provide comprehensive information on RCTs of CAM in Korea to those who could use this information when conducting English-language systematic reviews of traditional medicine therapies. The study aims to analyze the bibliometric characteristics and the risk of bias of RCTs on CAM in the Korean literature.

## 2. Methods

### 2.1. Searches

Eleven Korean bibliographic databases were searched electronically and sixteen Korean journals were searched manually, all from their inception to August 2013. Korean trials indexed in non-Korean databases such as MEDLINE or EMBASE were not considered. Key features of each database and journal searched are included in supplementary Appendix 1 in Supplementary Material available online at http://dx.doi.org/10.1155/2014/194047. For Korean trials of acupuncture, we updated the search carried out for our previous publication to August 2013 [10]. The CAM-related search terms used for searching electronic databases are included in supplementary Appendix 2. Korean language terms relevant to CAM, traditional medicine, and clinical trials were also used.

### 2.2. Study Selection

Two authors (CKK, DHK) conducted the literature search and retrieved citations of studies presumed to be potential RCTs. Full-text articles were obtained for all potentially eligible trials and two authors (CKK, BCS) independently checked the eligibility criteria of the full-text articles.

Parallel or crossover RCTs that assessed the efficacy or physiological features of complementary and traditional medicine were included, regardless of the participants' medical conditions, language (Korean or English) of publication, or publication year. Uncontrolled clinical trials and nonrandomized trials, including trials using a quasi-random method of allocation (e.g., alternation, date of birth, or case record number), were excluded.

We predefined types of CAM and grouped them according to the broad categories of natural products and mindbody medicine used by NCCAM. We included herbal medicine, ginseng, vitamin, and diet-based therapy in the natural products category and classified acupuncture, pharmacoacupuncture, qigong, magnet, tuina technique, moxibustion therapy, massage, taping technique, cupping therapy, meditation, tai-chi, yoga, and aromatherapy as mind and body practices [2].

Controls included placebo/sham, no treatment, or other active interventions. We included RCTs in which cointerventions were combined with CAM in the experimental group if the cointerventions were also given equally to the control group(s). However, if the purpose of the trial was to assess the efficacy of the cointervention and not the CAM therapy, the trial was excluded. All types of participants, even healthy subjects, were included because our research aim was to present all of the features of CAM RCTs in the Korean literature.

### 2.3. Data Extraction and Analysis

The characteristics of the included RCTs were analyzed and extracted in the following categories: (1) publication year and journal for bibliometric analysis; (2) study design and structure of CAM experimental and control groups; (3) medical conditions of participants; (4) sample size of total, experimental group and control group. The medical conditions of participants were categorized according to a revision of the International Classification of Disease 10 [14].

### 2.4. Risk of Bias

Risk of bias was assessed independently by two authors (CKK, DHK) using the Cochrane Collaboration's tool for assessing risk of bias, which includes judgments of “low,” “high,” or “unclear” risk of bias stemming from the following seven domains [15]: (1) random sequence generation; (2) allocation concealment; (3) blinding of participants and personnel; (4) blinding of outcome assessor; (5) incomplete outcome data; (6) selective reporting; and (7) other sources of bias. For “blinding of outcome assessor,” we considered the blinding relative to the primary outcome. For “incomplete outcome data,” we judged the risk of bias to be high if there was any missing outcome data for the primary outcome. For “selective reporting,” we judged the risk of bias to be low if all prespecified outcomes in a prepublished protocol were reported in the publication. We did not consider “other sources of bias.”

Disagreements were resolved by consensus through discussion between the two reviewers based on the judgment criteria in Chapter 8 of the* Cochrane Handbook for Systematic Reviews of Interventions* [14] and, if needed, by asking for further evaluation by a third reviewer.

## 3. Results

### 3.1. Study Description

As shown in supplementary Appendix S3, our searchers identified 2948 citations of which 553 full-text articles were evaluated in full and 193 full-text articles were excluded, leaving 360 trials that met our inclusion criteria. The literature search process is summarized in supplementary Appendix S3, following the PRISMA flow diagram.

### 3.2. Bibliometric Analysis

**Figure 1:**
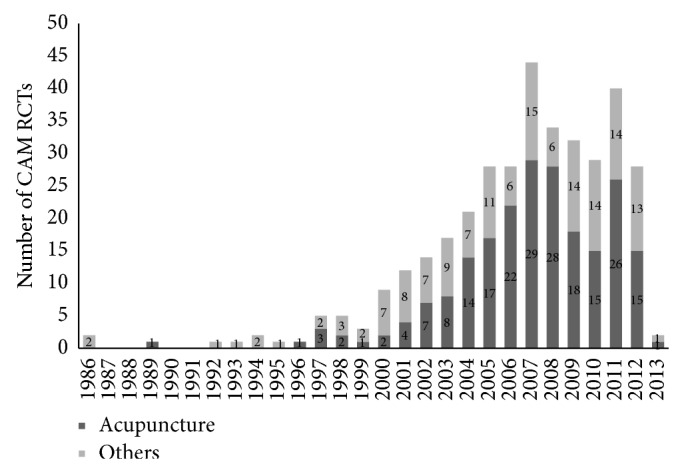
Number of complementary and traditional medicine randomized controlled trials in Korea by publication year.

The first CAM-related RCT that was published in Korea was in 1986 upon qigong and a second RCT that was published in 1989 was upon acupuncture. The third RCT that we found was a qigong RCT published in 1992. From the late 1990s, the total number of publications increased. From 2005 onwards, there is a steep increase in the number of RCTs on CAM, with the highest number of publications occurring in 2007 (44 RCTs). Since then, there has been a fluctuation in the number of publications per year, with 28 being published in 2012. The low number of RCTs in 2013 is due to the search covering only part of 2013 (Figure 1).

A total of 360 RCTs were published; 336 of the 360 records were published in journals and 24 studies were identified from dissertations. The journals publishing CAM-related RCTs were mostly related to traditional Korean medicine (23 of 52 journals, 282 of 360 papers; Table 1). All journals are shown in Appendix 4.

### 3.3. Study Design

The study designs are shown in Table 2. Parallel designs (*n* = 344; 95.6%) were more commonly used than crossover design RCTs (*n* = 16; 4.4%), with the two-arm parallel design predominating (*n* = 284, 78.9%).

### 3.4. Medical Conditions

Of the included trials, 71.1% were classified into one of 16 medical condition categories with the greatest concentrations being in the categories musculoskeletal conditions (25.8%), circulatory conditions (12.2%), nervous system conditions (6.1%), endocrine, nutritional and metabolic conditions (5.3%), and others (21.7%). The remaining 28.9% of trials were conducted in healthy participants (Table 3).

### 3.5. Sample Size

The total number of participants from the 360 included RCTs was 18,013. Sample size per trial ranged from 8 to 320 with a mean ± SD of 54.6 ± 48.7. The number of participants from (classic acupuncture (*n* = 144) or pharmacoacupuncture (*n* = 70)) acupuncture RCTs (*n* = 214) was 9,217, ginseng RCTs (*n* = 19) was 2,150, herbal medicine RCTs (*n* = 30) was 1,984, qigong and magnet RCTs (*n* = 31) was 1931, tuina technique RCTs (*n* = 17) was 728, moxibustion therapy RCTs (*n* = 12) was 459, taping RCTs (*n* = 7) was 364, massage RCTs (*n* = 17) was 354, meditation, tai-chi, yoga, and aroma therapy RCTs (*n* = 10) was 397, vitamin and diet-based therapy RCTs (*n* = 5) was 227, and cupping therapy RCTs (*n* = 5) was 202. The therapy with the greatest number of participants was acupuncture and the therapy with the smallest number was cupping therapy.

### 3.6. Risk of Bias

The risk of bias assessment for the included 360 RCTs is shown in Table 4. A random sequence was described as adequately generated in 41.7% (*n* = 150) of the included RCTs. Many studies did not specify how the random sequence was generated and they were estimated as unclear risk of bias. Only 8.3% of the RCTs (*n* = 30) described allocation concealment. The proportions of trials in which participants (patients) were blinded (*n* = 123; 34.2%), outcome assessors were blinded (*n* = 81; 22.5%), and incomplete outcome data were addressed (*n* = 160; 44.4%) were generally low. Very few trials were free of selective outcome reporting; only eight protocols (acupuncture (*n* = 3), herbal medicine (*n* = 2), and ginseng (*n* = 3)) were published, and each of these trials was determined to have a low risk of bias from selective outcome reporting.

## 4. Discussion

This study is an analysis of CAM RCTs published in the Korean literature. We found a substantial number of clinical trials on CAM in the Korean literature, especially acupuncture RCTs, even though Korean trials indexed in English-based databases were not considered in our search. The trials assessed the effectiveness of various types of CAM treatments, primarily related to traditional Korean medicine, in a variety of medical conditions. We found that RCTs of CAM in the Korean literature slowly emerged in the mid-1990s and the number of trials increased markedly in the mid-2000s. This is likely because a concept of guidelines for good clinical practice (GCP) for clinical trials by the Korean Food and Drug Administration (KFDA) was implemented in Korea in the late 1990s, and these guidelines had a great impact on clinical trials in Korea, beginning in the mid-2000s [16].

In a previous study documenting the types of CAM therapies included in a large database of international CAM controlled trials, the most common intervention type was nonvitamin, nonmineral dietary supplements (34%), followed by Chinese herbal medicines (27%) [5]. In comparison, we found that, in Korean CAM RCTs, the most common intervention type was acupuncture (59.4%), followed by Korean herbal medicine (8.3%), qigong (7.8%), and ginseng (5.3%).

The reason for the high percentage of acupuncture and herbal medicine is the unusual dual healthcare delivery system in Korea. Korea's National Health Insurance (NHI) provides coverage for traditional medicine such as acupuncture, herbal medicine, and cupping therapy as well as for conventional medical therapies [17]. A second reason for the high proportion of trials of acupuncture and herbal medicine is that the Korean government mainly funds research on acupuncture and herbal medicine. A likely reason for the large number of ginseng trials is that the Korea Tobacco & Ginseng (KT&G) Corporation funds ginseng research as part of its strategy for establishing the evidence for the effectiveness of its ginseng products. Additionally, there is an academic society, the Korean Society of Ginseng [18], which promotes ginseng research in Korea and publishes ginseng trials in its journal [19].

Although CAM RCTs in Korea have strength in quantity; on the whole, the high risk of bias remains their weakness. As mentioned above, a random allocation sequence was described as adequately generated in 41.7% of studies whereas only 8.3% described adequate allocation concealment. The overall proportions of trials reporting blinding of participants or personnel or blinding of outcome assessors were low; however, a comparatively larger proportion of herbal medicine and ginseng trials reported such blinding. This may be because many ginseng studies were funded by KT&G and tended to be of higher quality [20]. Furthermore, the majority of herbal medicine and ginseng trials were published after 2007. Recently published trials tended to show lower risk of bias, likely because reporting guidelines [21] have been developed and implemented, and reporting may have improved for more recent trials, allowing accurate assessment of trial procedures. Very few of the included RCTs (2.2%) were free of selective outcome reporting, due to the low number of published protocols.

Previous publications examining the quality of conventional medicine RCTs published in Korean medical journals found that in the Journal of Korean Medical Science (JKMS) 18% of trials reported adequate allocation concealment and 36% reported double blinding [22], in the Korean Journal of Urology (KJU) 86% of trials reported adequate randomization procedures, 4% reported adequate allocation concealment, and 62% reported double blinding [23], and in the Journal of the Korean Academy of Family Medicine (KAFM) 35% of trials reported adequate randomization procedures, 13% reported adequate allocation procedures, and 13% reported double blinding [24]. This result shows the similarity between RCTs of conventional medical and RCTs of CAM in the Korean literature.

There are some limitations to this study. First, in spite of our effort to retrieve all relevant articles, we cannot be certain that our search was all-inclusive. Unlike MEDLINE, no standard search filters for RCTs or complementary medicine exist in Korean databases, and therefore we had to review papers one by one; this study is a summary of Korean CAM that was completed via a manual search.

A great number of Korean databases and relevant journals were searched for this study, allowing the largest number of RCTs on CAM in the Korean literature to be evaluated. The number of articles related to intervention type or various medical conditions can be used for future research. Classified information from this study may be of help to future researchers.

## 5. Conclusions

This study has identified and evaluated the largest number of difficult to locate CAM RCTs in the Korean literature to date. These RCTs on CAM in the Korean literature have assessed the effectiveness of various types of CAM on a variety of medical conditions, reflecting the diverse application of CAM in Korean clinical practice. Although these RCTs have increased in number, there is great room for improvement in their methodological quality. It is encouraging that more recently published trials tended to show lower risk of bias. CAM systematic reviewers who cannot access Korean databases and journals might review the compiled list of these Korean RCTs (see supplementary Appendix S5) to identify any that are potentially eligible for their reviews.

## Supplementary Material

Appendix 1: 11 electronic databases and 16 manually searched Korean journals for literature search.
Appendix 2: Search terms for used electronic databases for literature search.
Appendix 3: PRISMA flow diagram of the whole literature search process.
Appendix 4: The number of complementary and traditional medicine randomised clinical trials in the Korean literature published by journal, respectively.
Appendix 5: Total list of complementary and traditional medicine RCTs included in bibliometric analysis and quality assessment.

## Figures and Tables

**Table 1:** Number of complementary and traditional medicine randomized controlled trials by journal published in the Korean literature *n* (%).

Publication field	Natural products			Mind and body practices									Total
	Herbal medicine	Ginseng	Vitamin diet-based therapy	Acupuncture	Pharmacoacupuncture	Qigong magnet	Tuina technique	Moxibustion therapy	Massage	Taping technique	Cupping therapy	Meditation tai-chi yoga aroma therapy	
Journal													
Traditional Korean medicine	30 (100.0)	14 (73.7)	2 (40.0)	122 (84.7)	67 (95.7)	6 (19.4)	17 (100.0)	11 (91.7)	2 (20.0)	4 (57.1)	5 (100.0)	2 (20.0)	282 (78.3)
Sports society						16 (51.6)							16 (4.4)
Nursing society						1 (3.2)		1 (8.3)	3 (30.0)	1 (14.3)		5 (50.0)	11 (3.1)
Conventional medicine		5 (26.3)		3 (2.1)					1 (10.0)				9 (2.5)
Physical therapy				3 (2.1)					1 (10.0)				4 (1.1)
Nutrition society			3 (60.0)										3 (0.8)
Others				1 (0.7)		3 (9.7)			3 (30.0)	2 (28.6)		2 (20.0)	11 (3.1)
Dissertation													
Master's thesis				11 (7.6)	3 (4.3)	2 (6.5)						1 (10.0)	17 (4.7)
Ph.D. thesis				4 (2.8)		3 (9.7)							7 (1.9)

Total													
Subtotal	30 (8.3)	19 (5.3)	5 (1.4)	144 (40.0)	70 (19.4)	31 (8.6)	17 (4.7)	12 (3.3)	10 (2.8)	7 (1.9)	5 (1.4)	10 (2.8)	360 (100.0)
Total	54 (15.0)			306 (85.0)									360 (100.0)

**Table 2:** Study design and types of interventions in complementary and traditional medicine randomized controlled trials in the Korean literature *n* (%).

Publication field	Natural products			Mind and body practices									Total (*n* = 360)
	Herbal medicine (*n* = 30)	Ginseng (*n* = 19)	Vitamin diet-based therapy (*n* = 5)	Acupuncture (*n* = 144)	Pharmacoacupuncture (*n* = 70)	Qigong magnet (*n* = 31)	Tuina technique (*n* = 17)	Moxibustion therapy (*n* = 12)	Massage (*n* = 10)	Taping technique (*n* = 7)	Cupping therapy (*n* = 5)	Meditation tai-chi yoga aroma therapy (*n* = 10)	
Parallel design													
Parallel, 2 arms	25 (83.3)	10 (52.6)	3 (60.0)	101 (70.1)	59 (84.3)	29 (93.5)	16 (94.1)	12 (100.0)	8 (80.0)	7 (100.0)	5 (100.0)	9 (90.0)	284 (78.9)
Parallel, 3 arms	4 (13.3)	2 (10.5)		25 (17.4)	9 (12.9)	2 (6.5)	1 (5.9)		2 (20.0)			1 (10.0)	46 (12.8)
Parallel, 4 arms		1 (5.3)	2 (40.0)	4 (2.8)	1 (1.4)								8 (2.2)
Parallel, 5 arms		4 (21.1)		1 (0.7)									5 (1.4)
Parallel, 6 arms				1 (0.7)									1 (0.3)
Crossover design	1 (3.3)	2 (10.5)		12 (8.3)	1 (1.4)								16 (4.4)

**Table 3:** Categories of medical conditions in complementary and traditional medicine randomized controlled trials in the Korean literature *n* (%).

Publication field	Natural products			Mind and body practices									Total
	Herbal medicine	Ginseng	Vitamin diet-based therapy	Acupuncture	Pharmacoacupuncture	Qigong magnet	Tuina technique	Moxibustion therapy	Massage	Taping technique	Cupping therapy	Meditation tai-chi yoga aroma therapy	
Musculoskeletal conditions	**1 (3.3)**			**40 (27.8)**	**33 (47.1)**	**3 (9.7)**	**5 (29.4)**	**3 (25.0)**		**6 (85.7)**	**2 (40.0)**		**93 (25.8)**
Low back pain				13	10		1			3	1		
Shoulder pain				7	1			1		1	1		
Sprain (ankle, wrist)				7	7		1			1			
Knee osteoarthritis	1			7	8	1		2					
Neck pain				2	5	1	1			1			
Myalgia				2	1	1							
Elbow joint pain				1	1								
Axial spondyloarthritis				1									
Temporomandibular joint syndrome							2						
Circulatory conditions	**4 (13.3)**	**2 (10.5)**		**17 (11.8)**	**9 (12.9)**		**4 (23.5)**	**5 (41.7)**			**1 (20.0)**	**2 (20.0)**	**44 (12.2)**
Stroke	2			15	9		4	4			1	1	
Hypertensive disease	1	1		2				1				1	
Heart diseases	1	1											
Nervous system conditions				**14 (9.7)**	**6 (8.6)**					**1 (14.3)**		**1 (10.0)**	**22 (6.1)**
Bell's palsy				5	5					1			
Headache				5	1							1	
Parkinson's disease				4									
Endocrine, nutritional, and metabolic conditions	**6 (20.0)**	**4 (21.4)**	**2 (40.0)**	**3 (2.1)**	**3 (4.3)**							**1 (10.0)**	**19 (5.3)**
Obesity	6	2	1	3	3							1	
Menopausal symptom		2	1										
Genitourinary conditions		**1 (5.3)**		**8 (5.6)**	**1 (1.4)**	**1 (3.2)**		**1 (8.3)**				**2 (20.0)**	**14 (3.9)**
Primary dysmenorrhea				4				1					
Hot flush		1		2									
Premenstrual tension syndrome				1	1	1						2	
Stress incontinence				1									
Motor or nonmotor vehicle accident				**3 (2.1)**	**4 (5.7)**		**8 (47.1)**				**1 (20.0)**		**16 (4.4)**
Mental and behavioral disorder		**2 (10.6)**		**6 (4.2)**								**1 (10.0)**	**9 (2.5)**
Unspecified mood disorder				4									
Anxiety disorder				1									
Tobacco use				1									
Alzheimer's disease		1											
Alcohol harmful use												1	
Impotence		1											
Digestive conditions	**1 (3.3)**	**1 (5.3)**		**3 (2.1)**	**1 (1.4)**			**1 (8.3)**	**2 (20.0)**				**9 (2.5)**
Dyspepsia	1			2	1								
Constipation				1				1	2				
Chronic gastritis		1											
Conditions of the skin and subcutaneous tissue	**6 (20.0)**			**2 (1.4)**									**8 (2.2)**
Atopic dermatitis	5												
Acne vulgaris	1			2									
Ear, nose, and throat conditions				**5 (3.5)**									**5 (1.4)**
Temporomandibular joint disorder				2									
Allergic rhinitis				2									
Tinnitus				1									
Neoplasms			**1 (20.0)**		**1 (1.4)**				**2 (20.0)**				**4 (1.1)**
Postprocedural disorders				**3 (2.1)**									**3 (0.8)**
Nausea, vomiting, and pain after surgery				3									
Symptoms, signs, and abnormal clinical and laboratory findings				**3 (2.1)**									**3 (0.8)**
Pregnancy, childbirth, and the puerperium	**1 (3.3)**				**1 (1.4)**	**1 (3.2)**							**3 (0.8)**
Eyes conditions		**1 (5.3)**							**1 (10.0)**				**2 (0.6)**
Respiratory condition	**2 (6.6)**												**2 (0.6)**
Healthy participants	**9 (30.0)**	**8 (42.1)**	**2 (40.0)**	**37 (25.7)**	**11 (15.7)**	**26 (83.9)**		**2 (16.7)**	**5 (50.0)**		**1 (20.0)**	**3 (30.0)**	**104 (28.9)**

Total	30	19	5	144	70	31	17	12	10	7	5	10	360

**Table 4:** Methodological quality of complementary and traditional medicine RCTs in Korea according to Cochrane tool for assessing risk of bias *n* (%)^*^.

Risk of bias	Natural products			Mind and body practices									Total (*n* = 360)
	Herbal medicine (*n* = 30)	Ginseng therapy (*n* = 19)	Vitamin diet-based therapy (*n* = 5)	Acupuncture (*n* = 144)	Pharmacoacupuncture (*n* = 70)	Qigong magnet (*n* = 31)	Tuina technique (*n* = 17)	Moxibustion therapy (*n* = 12)	Massage (*n* = 10)	Taping technique (*n* = 7)	Cupping therapy (*n* = 5)	Meditation tai-chi yoga aroma therapy (*n* = 10)	
(1) Random sequence generation	18 (60.0)	11 (57.9)	1 (20.0)	65 (45.1)	34 (48.6)	2 (6.5)	5 (29.4)	4 (33.3)	3 (30.0)	3 (42.9)	1 (20.0)	3 (30.0)	150 (41.7)
(2) Allocation concealment	3 (10.0)	7 (36.8)	0	17 (11.8)	3 (4.3)	0	0	0	0	0	0	0	30 (8.3)
(3) Blinding of participants and personnel													
Patients	23 (76.7)	17 (89.5)	3 (60.0)	47 (32.6)	30 (42.9)	4 (12.9)	0	1 (8.3)	1 (10.0)	1 (14.3)	0	0	123 (34.2)
Doctor	22 (73.3)	16 (84.2)	3 (60.0)		18 (25.7)								59 (16.4)
(4) Blinding of outcome assessment	22 (73.3)	14 (73.7)	3 (60.0)	27 (18.8)	8 (11.4)	3 (9.7)	0	0	2 (20.0)	1 (14.3)	1 (20.0)	0	81 (22.5)
(5) Incomplete outcome data	27 (90.0)	15 (78.9)	5 (100.0)	42 (29.2)	30 (42.9)	9 (29.0)	2 (11.8)	9 (75.0)	5 (50.0)	7 (100.0)	3 (60.0)	6 (60.0)	160 (44.4)
(6) Selective reporting	2 (6.7)	3 (15.8)	0	3 (2.1)	0	0	0	0	0	0	0	0	8 (2.2)

^*^Low risk of bias, *n* (%).
