# Supplementary material for: Randomized Controlled Trials on Complementary and Traditional Medicine in the Korean Literature
Source: Evid Based Complement Alternat Med. 2014 Dec 24;2014:194047. doi: 10.1155/2014/194047 (PMC4290637; doi:10.1155/2014/194047)
Supplement: Supplementary file 1 — Appendix 1: 11 electronic databases and 16 manually searched Korean journals for literature search. Appendix 2: Search terms for used electronic databases for literature search. Appendix 3: PRISMA flow diagram of the whole literature search process. Appendix 4: The number of complementary and traditional medicine randomised clinical trials in the Korean literature published by journal, respectively. Appendix 5: Total list of complementary and traditional medicine RCTs included in bibliometric analysis and quality assessment. [file 194047.f1.pdf]

Appendix 1. Electronic databases and manually searched journals for literature search

Title	URL	Feature
**Electronic database**		
National Assembly Library	www.nanet.go.kr	Books, articles, dissertations, electronic data, etc. in all fields
Research Information Sharing Service	www.riss4u.net	Journals articles and dissertations of nationwide universities
Korean Studies Information Service System	kiss.kstudy.com	Domestic journal articles in all fields
DBPIA	www.dbpia.co.kr	Domestic journal articles in all fields
Korean Medical Database	kmbase.medric.or.kr	Domestic journal articles in health and medical fields
KoreaMed	www.koreamed.org	Bibliography and abstract of domestic medical journals in English
Korea Institute of Science and Technology Information	www.kisti.re.kr	Domestic and international journal articles in science and technology
OASIS	oasis.kiom.re.kr	Journal articles and statistical reports in Korean medicine
National Digital Library	www.dlibrary.go.kr	Integrated linkage of main libraries and databases of Korea

Korean Traditional Knowledge Portal	www.koreantk.com	Articles and information in Korean medical field
Research Information Center for Health	www.richis.org	Articles of 90 journals in nursing or health
**Journal**		
The Journal of Korean Acupuncture & Moxibustion Society	www.acumoxa.or.kr	Articles related to Korean acupuncture and moxibustion
Journal of Oriental Rehabilitation Medicine	www.ormkorea.org	Articles related to Korean rehabilitation medicine
Journal of Korean Oriental Medical Society	www.koms.or.kr	Articles in all fields of Korean medicine
Journal of Meridian & Acupoint	www.acupoint.org	Articles related to meridian & acupuncture point
The Journal of Korea CHUNA Manual Medicine for Spine & Nerves	www.chuna.or.kr	Articles related to *Chuna* manipulation
Korean Journal of Oriental Physiology & Pathology	www.ksomp.or.kr	Articles related to Oriental Physiology & Pathology
The Journal of Oriental Obstetrics & Gynecology	www.oobgy.or.kr	Articles related to Oriental Obstetrics & Gynecology
Journal of Ginseng Research	www.ginsengres.org	Articles related toGinseng therapy
Journal of Korean Oriental Internal Medicine	www.oim.or.kr	Articles related to Korean internal medicine

The Korea Association of Herbology	www.herbology.or.kr	Articles related to Herbal medicine
The Journal of Korean Academy of Medical Gi-gong	www.gigong.or.kr	Articles related to medical gi-gong
Korean Academy of University Trained Physical Therapists	www.kosombe.or.kr	Articles related to physical therapy
The Korean Academy of Physical Therapy Science	www.kpt.or.kr	Articles related to physical therapy
Journal of Biomedical Engineering Research	www.kosombe.or.k	Articles related to biomedical engineering
Journal of Pharmacopuncture	www.pharmacopuncture.co.kr	Articles related to pharmacopuncture
The Korean Pain Society	www.painfree.or.kr	Articles related to pain

Appendix 2. Search term for used electronic databases

CAM category	SEARCH TERM USED FOR ELECTRONIC DATABASE
**1. Natural Products**	
Herbal medicine	'(herbal medicine OR herb OR herbal) AND (randomized OR controlled)'
Ginseng therapy	'(ginseng) AND (randomized OR controlled)'
Vitamin	'(vitamin) AND (randomized OR controlled)'
Diet-based therapy	'(diet OR biological) AND (randomized OR controlled)'
**2. Mind and Body Practices**	
Acupuncture	'(acupuncture OR acupuncture point OR meridian) AND (random OR control)'
Pharmaco acupuncture	'(Pharmaco acupuncture OR Pharmaco acup* OR meridian) AND (random OR control)'
Qigong	'(energy therapy OR qigong OR training) AND (randomized OR controlled)'
Magnet	'(magnet) AND (randomized OR controlled)'
Tuina technique	'(tunia OR chuna) AND (randomized OR controlled)'
Moxibustion therapy	'(moxibustion) AND (random OR control)'
Massage	'(massage) AND (randomized OR controlled)'
Taping techinque	'(taping) AND (randomized OR controlled)'
Cupping therapy	'(cupping) AND (random OR control)'
Meditation	'(meditation OR mind-body) AND (randomized OR controlled)'
Yoga	'(yoga) AND (randomized OR controlled)'
Aroma therapy	'(aroma) AND (randomized OR controlled)'
Tai chi	'(tai-chi) AND (randomized OR controlled)'

Appendix3. PRISMA flow diagram of the literature search

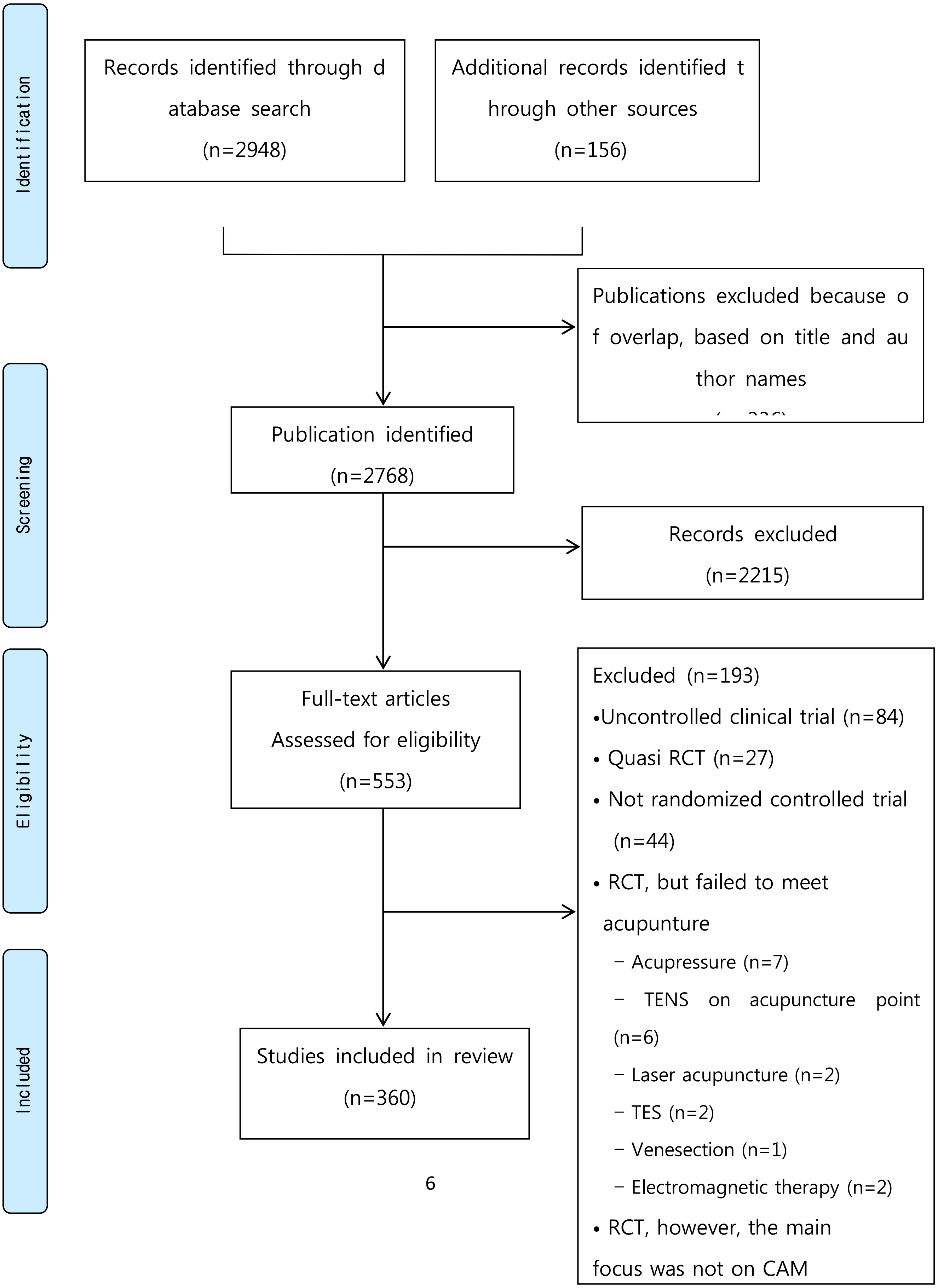

Appendix 4. Number of complementary and traditional medicine randomised clinical trials in the Korean literature published by journal.

Journal field	Natural Products			Mind and Body Practices									Total
	Herbal medicine	Ginseng therapy	Vitamin diet-based therapy	Acupuncture	Pharmaco acupuncture	Qigong Magnet	Tuina technique	Moxibustion therapy	Massage	Taping technique	Cupping therapy	Meditation Tai chi Yoga Aroma therapy	
**Traditional Korean medicine**	**30**	**14**	**2**	**122**	**67**	**6**	**17**	**11**	**2**	**4**	**5**	**2**	**282**
The Journal of Korean Acupuncture and Moxibustion society				64	30		2	3		1	1		101
Journal of Oriental Rehabilitation Medicine	3		1	19	6	3	8			2	2	1	45
Korean Pharmacopuncture institute					24								24
Journal of Korean Oriental Medical Society	6			8	1			2		1	2		20
Journal of Korean Oriental Internal Medicine	7			5	1			2				1	16
Journal of Meridian and Acupoint				10	1								11
The Journal of Korea Chuna Manual Medicine for Spine and Nerves				3	1		6						10
Korean Journal of Oriental Physiology & Pathology	4	3		1				1					9
The Journal of Oriental Obstetrics and Gynecology	1	1		4	2			1					9
Journal of Ginseng Research		7											7

	Journal of Oriental Neuropsychiatry				6	1								7
	The Korean Oriental Medical Ophthalmology & Otolaryngology & Dermatology Society	5								1				6
	Society of Korean Medicine for Obesity Research	2	1											3
	The Journal of Korean Academy of Medical Gi-gong						3							3
	The Korea Association of Herbology	1	1											2
	The Korean Society for Oriental Preventive Medicine								2					2
	Korean Journal of Oriental Medicine				1									1
	Journal of Oriental Medicine				1									1
	The Korean Journal Of Oriental Medical Prescription	1												1
	Society of Sports Korean Medicine							1						1
	Korean Pharmacopuncture institute									1				1
	Ginseng society		1											1
	Traditional Medicine Research of Deajeon University			1										1
**Sports society**							**16**							**16**
	Korea Sports Research						7							7
	Korean Society of Sport And Leisure Studies						4							4

Sciences of Physical education papers						3							3
Korean Society of Sport Psychology						1							1
The Korean Society of Sports Medicine						1							1
**Nursing**						**1**		**1**	**3**	**1**		**5**	**11**
Korean Society of Nursing Science						1			1			2	4
Korean Academy of Fundamentals of Nursing								1				1	2
Korean Society of Adult Nursing									1	1			2
The Korean Academy of Psychiatric and Mental Health Nursing												1	1
Journal of Korean Academy of Rural Health Nursing									1				1
Korean Society of Biological Nursing Science												1	1
**Conventional medicine**		**5**		**3**					**1**				**9**
The Journal of Korean Academy of Craniomandibular				1									1
Korean Journal of Urology		1											1
The Korean Association of Internal Medicine		1											1
Menopause		1											1
Sleep medicine and psychophysiology		1											1
The Korean Prostation Society		1											1
The Korean Pain Society				1									1

Korean Society For Hospice And Palliative Care									1				1
The Korean Journal of Thoracic and Cardiovascular Surgery				1									1
**Physical therapy**				**3**					**1**				**4**
The Korean Academy of Physical Therapy Science				2					1				3
Korean Academy of University Trained Physical Therapists				1									1
**Nutrition society**			**3**										**3**
The Korean Society of Food Science and Nutrition			1										1
The Korean journal of Nutirition			2										2
**Others**				**1**		**3**			**3**	**2**		**2**	**11**
The Korea Academia-Industrial cooperation Society									1	2		1	4
The Korean Society of Jungshin Science						3							3
Journal of Biomedical Engineering Research				1					1				2
Korean Society for Emotion & Sensibility												1	1
Korea Coaching Development Research									1				1
**Studies not published in journals**				**15**	**3**	**5**						**1**	**24**
Masterʹs thesis				11	3	2						1	17
PhD thesis				4		3							7

total	30	19	5	144	70	31	17	12	10	7	5	10	360

Appendix 5. Complementary and Traditional medicine RCTs included in bibliometric analysis and quality assessment (N = 360)

Randomized Clinical Trials on Acupuncture in Korean Literature:

Bibliometric Analysis and Methodological Quality (N=144)

No	Included article
1	C. W. Seung, "Clinical Report on the Comparing with Acupuncture and Herb-Decoction Treatment of Aural Vertigo Causes by Phlegm and Dampness [in English]," *The Journal of Korean Acupuncture & Moxibustion Society*, vol. 6, no. 1, pp. 41-50, 1989.
2	Y. S. Kim, C. H. Kim, and K. S. Kim, "Effect of Auricular Acupuncture on Postoperative Nausea and Vomiting [in English]," *The Journal of Korean Oriental Medical Society*, vol. 17, no. 2, pp. 331-336, 1996.
3	K. S. Cho, S. C. Kim, J. Y. Lee, S. S. Sohn, and D. S. Park, "The Effect of Acupuncture on the Post-Thoracotomy Pain Control [in Korean]," *The Korean Journal of Thoracic and Cardiovascular Surgery*, vol. 30, no. 2, pp. 187-194, 1997.
4	H. C. Ko, W. H. Cho, and S. S. Han, "Effect of Microcurrent Electrical Neuromuscular Stimulation of Auricular and Somatic Acupuncture Points on Experimental Pressure Threshold [in Korean]," *Physical Therapy Korea*, vol. 4, no. 2, pp. 36-50, 1997.
5	S. H. Lee, and Y. H. Lee, "Clinical Study with Thermography on Shoulder Hand Syndrome after Stroke [in Korean]," *The Journal of Korean Oriental Medical Society*, vol. 18, no. 1, pp. 25-39, 1997.
6	S. R. Cheon, and E. S. Lee, "The Effects of the Magnetic Therapy Treated on Acupuncture Points for Recovery from Fatigue with before and after Exercise [in Korean]," *Journal of Oriental Rehabilitation Medicine*, vol. 8, no. 1, pp. 158-170, 1998.
7	J. C. Seo, W. J. Chung, J. H. Kim, et al., "The Effect of Cranial and Penetrational Acupuncture on Recovery of Motor Disorder due to Stroke [in English]," *Journal of Oriental Medicine*, vol. 3, no. 1, pp. 19-27, 1998.
8	T. S. Park, J. S. Park, and M. Y. Ko, "A Clinical Effect of Electro-Acupuncture Stimulation Therapy on TMD Patients [in English]," *The Journal of Korean Academy of Craniomandibular Disorders*, vol. 11, no. 2, pp. 103-111, 1999.
9	Y. S. Kim, "Antispastic Effects of Electroacupuncture, TENS and NMES in Stroke Patient [in Korean]," *The Journal of Korean Acupuncture & Moxibustion Society*, vol. 17, no. 2, pp. 209-220, 2000.
10	W. K. You, and M. J. Lee, "The Effects of Auricular Acupuncture for Obesity on the Change of Hormone and Energy Metabolism during Weight Control of Veteran Taekwondo Players [in Korean]," *Journal of Oriental Rehabilitation Medicine*, vol. 10,

no. 1, pp. 133-145, 2000.

11 S. J. Chae, N. O. Kim, Y. C. Park and S. S. Son, "Comparison of the Improvement of Subjective Symptoms between Body Acupuncture Group & 8 Constitution Acupuncture Group [in Korean]," *The Journal of Korean Acupuncture & Moxibustion Society*, vol. 18, no. 3, pp. 48-55, 2001.

12 S. I. Kang, C. W. Lim, W. T. Park, W. R. So, M. J. Song, and Y. S. Kim, "The Clinical study of acupuncture and moxibustion therapy on ch'ŏnch'u(ST25) for constipation [in Korean]," *The Journal of Korean Acupuncture & Moxibustion Society*, vol. 18, no. 6, pp. 125-134, 2001.

13 S. H. Ryu, "Effect of Electroacupuncture Stimulation on the Hemiplegic Upper Extremity after Stroke [in Korean]," *Master's thesis: KyungHee Univeristy*, 2001.

14 S. M. Ryu, J. S. Hwang, and E. K. Rhu, "The Clinical Effect on Low Back Pain by Young-gol and Dae-back Acupuncture Points [in Korean]," *Journal of Oriental Rehabilitation Medicine*, vol. 12, no. 4, pp. 1-10, 2001.

15 B. S. Chang, K. S. Jin, J. W. Kim, et al., "Clinical Study on The Remedial Effect of Oriental Medicine Used EAV(Meridian) [in Korean]," *The Journal of Korean Acupuncture & Moxibustion Society*, vol. 19, no. 6, pp. 80-96, 2002.

16 J. S. Hwang, S. M. Ryu, and E. K. Rhu, "The Clinical Study on the Dong-Si Acupuncture Treatment at the Acute Ankle Sprain [in Korean]," *Journal of Oriental Rehabilitation Medicine*, vol. 12, no. 3, pp. 123-129, 2002.

17 H. B. Joung, and S. B. Bang, "Effects of Electrotherapy on the Deactivation of Trigger points [in Korean]," *The Korean Academy of Physical Therapy Science*, vol. 9, no. 2, pp. 123-131, 2002.

Randomized Clinical Trials on Acupuncture in Korean Literature:

Bibliometric Analysis and Methodological Quality

No	Included article
18	K. O. Min, S. H. Kim, and S. J. Park, "The Effect of the Needle Electrode Electrical Stimulation in the Auricular Therapy on the smokers and non-smokers with Low Back Pain [in Korean]," *The Korean Academy of Physical Therapy Science*, vol. 9, no. 1, pp. 45-52, 2002.
19	S. H. Ryu, K. S. Lee, T. K. Kim, et al., "Effects of Electroacupuncture on the Hemiplegic Upper Extremity after Stroke [in Korean]," *The Journal of Korean Oriental Medical Society*, vol. 23, no. 2, pp. 180-189, 2002.
20	D. Y. Choi, D. I. Lee, S. Y. Kim, K. S. Kim, J. D. Lee, and Y. H. Lee, "Specific Electric

Acupuncture for the Management of Central Poststroke Pain [in Korean]," *The Korean Pain Society*, vol. 13, no. 1, pp. 74-80, 2003.

21 S. J. Kim, M. S. Kim, D. Y. Son, et al., "Effects of Acupuncture Stimulation on Current Perception Threshold in Healthy Adults : Single-blind, Randomized Controlled Trial [in Korean]," *Journal of Oriental Rehabilitation Medicine*, vol. 13, no. 3, pp. 29-38, 2003.

22 S. U. Kim, J. S. Lee, S. S. Kim, H. D. Shin, and S. H. Chung, "The Effect of Microcurrent Electrical Neuromuscular Stimulation on Stress-related Hormones [in Korean]," *Journal of Oriental Rehabilitation Medicine*, vol. 13, no. 4, pp. 1-18, 2003.

23 K. M. Lee, S. Y. Lee, S. W. Kim, et al., "Is It possible To Apply Placebo Auricular Acupuncture to Korean? [in Korean]," *The Journal of Korean Acupuncture & Moxibustion Society*, vol. 20, no. 2, pp. 145-160, 2003.

24 W. J. Sim, S. H. Jung, and S. S. Kim, , "Specific Electric Acupuncture for the Management of Central Poststroke Pain [in Korean]," *The Journal of Oriental Rehabilitation Medicine*, vol. 13, no. 1, pp. 95-111, 2003.

25 S. J. Chae, and H. S. Song "The Effect of 8 Constitution Acupuncture on degenerative arthritis of knee joint [in Korean]," *Journal of Korean Acupuncture & Moxibustion Society*, vol. 21, no. 4, pp. 65-73, 2004.

26 I. T. Jung, S. Y. Kim, K. S. Kim, et al., "A clinical study of Aroma Acupuncture on chromic headache patients [in Korean]," *Journal of Korean Acupuncture & Moxibustion Society*, vol. 21, no. 5, pp. 123-136, 2004.

27 S. Kwak, W. J. Choi, S. G. Lee, and K. M. Park, "The Effects of Acupuncture Stimulation and Progressive Relaxation Therapy on Examination Stress of Students [in Korean]," *The Korean Journal of Meridian & Acupoint*, vol. 21, no. 2, pp. 161-176, 2004.

28 C. W. Lee, I. B. Park, S. W. Kim, et al., "The Effect of Acupuncture and Dong's Acupuncture about Bell'palsy [in Korean]," *Journal of Korean Acupuncture & Moxibustion Society*, vol. 21, no. 2, pp. 287-300, 2004.

29 S. H. Lee, E. J. Kim, S. P. Yun, et al., "The Effect of Intradermal Acupuncture on The Patients suffering from Insomnia after Stroke [in Korean]," *Journal of Korean Oriental Internal Medicine*, vol. 25, no. 1, pp. 138-148, 2004.

30 S. C. Lim, J. C. Seo, K. U. Kim, et al., "The Comparison Of Acupuncture Sensation Index Among Three Different Acupuncture Devices [in Korean]," *Journal of Korean Acupuncture & Moxibustion Society*, vol. 21, no. 6, pp. 209-219, 2004.

31 J. S. Park, W. Y. Kim, S. T. Baek, S. D. Lee and K. S. Kim, "Comparison of Superficial and Deep Acupuncture in the Treatment of Ankle sprain : A Randomized Controlled Trial-Pilot study [in Korean]," *Journal of Korean Acupuncture & Moxibustion Society*,

vol. 21, no. 5, pp. 137-147, 2004.

32 B. J. An, and H. S. Song, "Effect of Electroacupuncture on Patients with Peripheral Facial Paralysis [in Korean]," *Journal of Korean Acupuncture & Moxibustion Society*, vol. 22, no. 4, pp. 121-129, 2005.

33 D. T. Choi, S. Lim, M. H. Cha, et al., "Effect on pain behavior in non-medicinal treatment applied to chronic headache patients [in Korean]," *The Korean Journal of Meridian & Acupoint*, vol. 22, no. 1, pp. 55-66, 2005.

34 M. B. Kim, H. D. Shin, and S. S. Kim, "The Influences of Electroacupuncture at Interosseous Muscle for Hand Function in Hemiplegic Patients after Stroke [in Korean]," *The Journal of Oriental Rehabilitation Medicine*, vol. 15, no. 4, pp. 17-28, 2005.

Randomized Clinical Trials on Acupuncture in Korean Literature:

Bibliometric Analysis and Methodological Quality

No	Included article
35	S. C. Kim, S. N. Kim, J. A. Lim, et al., "Effect of Acupuncture Treatment on the Premenstrual Symdrome : Controlled Clinical Trial [in Korean]," *Journal of Korean Acupuncture & Moxibustion Society*, vol. 22, no. 1, pp. 41-60, 2005.
36	S. K. Ko, H. G. Song, E. M. Kim, et al., "The Effect of Acupuncture Treatment on the Lumbar Extensor Muscle Flexibility [in Korean]," *Journal of Korean Acupuncture & Moxibustion Society*, vol. 22, no. 5, pp. 79-90, 2005.
37	H. S. Lee, J. S. Lee, and S. S. Kim, "The Comparison of Effectiveness in Electroacupuncture between Dong-si Acupoint and Body Acupoint in Hemiplegic Patients after Acute Stroke [in Korean]," *The Journal of Oriental Rehabilitation Medicine*, vol. 15, no. 4, pp. 55-64, 2005.
38	J. H. Lee, "The effect of combined treatment with ultrasound and electro-lipolysis acupuncture on the change of figure in obese women [in Korean]," *Master's thesis: YongIn University*, 2005
39	J. W. Lee, "A Randomized Controlled Trial of Acupuncture Effect of Kuesu-point(快兪穴) on Lower back pain and sciatica [in Korean]," *Master's thesis: DongGuk University*, 2005.
40	K. H. Lee, M. S. Kang, and H. S. Song, "A Clinical Study on the Effects of Acupuncture at *Sang-Baek* for Patients with Sprain of the Wrist Joint [in Korean]," *The Journal of Korean Acupuncture & Moxibustion Society*, vol. 22, no. 6, pp. 211-217, 2005.

41 Y. C. Park, J. H. Jo, K. E. Hong, W. C. Kang, and S. M. Choi, "Effect of Acupuncture on Nasal Obstruction in Patients with Persistent Allergic Rhinitis: A Randomized Controlled Trial [in Korean]," *The Journal of Korean Acupuncture & Moxibustion Society*, vol. 22, no. 6, pp. 229-239, 2005.

42 S. S. Yuk, and E. M. Lim, "A Clinical Study on the Effect of Crossing over Treatment of Acupuncture and Herbal Medication for Primary Dysmenorrhea [in Korean]," *The Journal of Oriental Obstetrics & Gynecology*, vol. 18, no. 4, pp. 144-152, 2005.

43 J. H. Cho, S. H. Chung, and S. S. Kim, "The Effect of Dong-Si Acupoint on the Meridian Muscle Tension of Governer Vessel and Blader Meridian [in Korean]," *Journal of Oriental Rehabilitation Medicine*, vol. 16, no. 3, pp. 83-92, 2006.

44 J. H. Cho, S. H. Chung, J. S. Lee, and S. S. Kim, "MEMG Analysis on Antispastic Effect of Electroacupuncture and Transcutaneous Electrical Nerve Stimulation [in Korean]," *The Journal of Oriental Rehabilitation Medicine*, vol. 16, no. 2, pp. 131-143, 2006.

45 J. C. Jung, K. H. Kim, Y. C. Park, et al., "The Study on the Effect of Acupuncture on UPDRS and Heart Rate Variability in the Patients with Idiopathic Parkinson's Disease [in Korean]," *Journal of Korean Acupuncture & Moxibustion Society*, vol. 23, no. 3, pp. 143-153, 2006.

46 W. S. Jung, J. W. Hong, J. W. Lee, et al., "The Clinical Effect of Manipulation of Acupuncture to *Shen-Men* and *Nei-Kuan* on Blood Pressure of Normal Male Subjects [in Korean]" *Korean Journal of Oriental Medicine*, vol. 27, no. 4, pp. 57-61, 2006.

47 D. H. Kim, D. H. Yang, E. J. Kim, et al., "A Study on the Effect of Interaction between Acupuncture at Shinmun(HT7) and Subject's Cold or Heat Tendency on Heart Rate Variability [in Korean]," *Journal of Korean Acupuncture & Moxibustion Society*, vol. 23, no. 1, pp. 25-38, 2006.

48 S. C. Kim, J. A. Lim, J. D. Lee, et al., "A Pilot Study of Acupuncture Treatment for the Osteoarthritis of the Knee Joint on the EBM (Evidence Basement Medicine) [in Korean]," *Journal of Korean Acupuncture & Moxibustion Society*, vol. 23, no. 1, pp. 187-215, 2006.

49 T. S. Kim, C. Y. Kim, K. H. Lee, T. W. Um, Y. H. Jung, and Y. T. Kho, "Comparative Clinical Study between the Acupuncture Treatment and the Chuna Treatment on Temporomandibular Disorder [in Korean]," *The Journal of Korea CHUNA Manual Medicine for Spine & Nerves*, vol. 1, no. 1, 2006.

50 H. Lee, K. E. Hong, Y. I. Kim, et al., "A Clinical Trial of Acupuncture Treatment for Frozen Shoulder [in Korean]," *Journal of Korean Acupuncture & Moxibustion Society*, vol. 23, no. 1, pp. 165-177, 2006.

51 S. Y. Lee, J. A. Lim, W. M. Na, C. S. Lee, D. J. Kim, and S. C. Kim, "The Study of the *Kim Sham* Acupuncture for Simgle Blind about the Acupuncture Points used for Treatment of the Shoulder Pain [in Korean]," *The Journal of Korean Acupuncture & Moxibustion Society*, vol. 23, no. 6, pp. 133-143, 2006.

Randomized Clinical Trials on Acupuncture in Korean Literature:

Bibliometric Analysis and Methodological Quality

No	Included article
52	D. W. Nam, H. B. Kim, D. H. Yang, et al., "Comparison Research of Clinical Effect of Eastern and Western Medical Treatment on Frozen Soulder Patients [in Korean]," *Journal of Korean Acupuncture & Moxibustion Society*, vol. 23, no. 5, pp. 105-113, 2006.
53	D. W. Nam, I. T. Jung, J. H. Kim, et al., "Clinical Observation of Western Medical Treatment and Acupuncture Treatment on Frozen Soulder Patients [in Korean]," *Journal of Korean Acupuncture & Moxibustion Society*, vol. 23, no. 5, pp. 177-185, 2006.
54	K. H. Rheu, I. H. Im, D. H. Kim, et al., "Effect of Acupuncture at PC6(Neiguan) and SP4(Gongsun) points on Autonomic Nervous System in Healthy Adults [in Korean]," *Journal of Korean Oriental Internal Medicine*, vol. 27, no. 3, pp. 703-710, 2006.
55	H. J. Ryu, and S. S. Kim, "Effect of Electoracupuncture by Different Insertion Method on Upper Limb Function in Post Stroke Patients with Hemiplegia [in Korean]," *The Journal of Oriental Rehabilitation Medicine*, vol. 16, no. 1, pp. 49-61, 2006.
56	S. C. Kim, E. H. Jang, W. M. Na, et al., "A Pilot Study of *Sa-am* Acupuncture Treatment Used by Sham Acupuncture for the Simple Obesity [in Korean]," *The Journal of Korean Acupuncture & Moxibustion Society*, vol. 24, no. 5, pp. 67-88, 2007.
57	D. I. Kim, J. J. Roh, S. K. Jung, M. S. Choi, and S. M. Choi, "A study on the minimal acupuncture using a modified guide tube for Korean female participants familiar with acupuncture therapy [in Korean]," *The Journal of Oriental Obstetrics & Gynecology*, vol. 20, no. 3, pp. 200-212, 2007
58	S. H. Kim, H. J. Park, H. A. Park, J. H. Jang, K. S. Hwang, and S. Y. Lee, "The Clinical Study on the Effect of *SAAM* Acupuncture Treatment for Patients with Fatigue [in Korean]," *The Journal of Korean Acupuncture & Moxibustion Society*, vol. 24, no. 6, pp. 149-157, 2007.
59	Y. C. Park, J. H. Jo, C. G. Son, et al., "Effect of Acupuncture Treatment for Functional Dyspepsia : A Randomized Controlled Trial [in Korean]," *The Journal of Korean Acupuncture & Moxibustion Society*, vol. 24, no. 1, pp. 1-12, 2007.
60	I. C. Jung, S. R. Lee, Y. C. Park, et al., "The Effect of Sa-am Acupuncture Treatment for Major Symptom of Hwa-byung : A Preliminary Study [in Korean]," *The Journal of*

Oriental Neuropsychiatry, vol. 18, no. 1, pp. 79-94, 2007.

61 H. Byun, S. W. Kim, J. H. Ahn, et al., "Individualized Acupuncture versus Standardized Acupuncture in Symptomatic Treatment for Osteoarthritis of the Knee-a Randomized Controlled Trial (ISRCTN 40706107) [in Korean]," *The Journal of Korean Acupuncture & Moxibustion Society*, vol. 24, no. 4, pp. 183-195, 2007.

62 J. S. Lee, K. H. Song, S. N. Lee, et al., "Clinical Study of Different Effect between Trigger Point Needling and Remote Acupuncture Point Needling on Shoulder Pain Patient [in Korean]," *The Journal of Korean Acupuncture & Moxibustion Society*, vol. 24, no. 5, pp. 89-96, 2007.

63 C. Y. Kim, N. H. Kwon, Y. J. Shin, et al., "Randomized Controlled Trial : Effect of Master *Dong*'s Acupuncture in Chronic Shoulder Pain Patients [in Korean]," *The Journal of Korean Acupuncture & Moxibustion Society*, vol. 24, no. 6, pp. 89-96, 2007.

64 D. W. Nam, Y. S. Choi, H. B. Kim, et al., "Randomized Controlled Trial of East-West Collaborate Medical Treatment on Female Chronic Shoulder Pain Patients [in English]," *The Journal of Korean Acupuncture & Moxibustion Society*, vol. 24, no. 6, pp. 113-122, 2007.

65 D. W. Nam, S. Lim, J. I. Kim, et al., "Clinical Observation of Acupuncture and Nerve Block Treatment for Adhesive Capsulitis Patients [in Korean]," *The Journal of Korean Acupuncture & Moxibustion Society*, vol. 24, no. 4, pp. 159-171, 2007.

66 Y. D. Kwon, S. G. Lee, C. W. Lee, S. K. Jung, D. E. Kim, and S. M. Choi, "The Short-term Efficacy of Acupuncture for Chronic Low Back Pain: Randomized Sham Controlled Trial [in English]," *The Journal of Oriental Rehabilitation Medicine*, vol. 17, no. 2, pp. 123-132, 2007.

67 D. J. Kim, Y. J. Choi, D. H. Kim, et al., "The Comparison of Effectiveness between Near Acupuncture Point Needing and Remote Acupuncture Point Needing on Treating Ankle Sprain of Acute Stage [in Korean]," *The Journal of Korean Acupuncture & Moxibustion Society*, vol. 24, no. 4, pp. 25-33, 2007.

68 K. E. Hong, Y. C. Park, J. H. Jo, et al., "Effect of Sa-am Acupuncture Method for Chronic Tension-type Headache: A Randomized Controlled Trial [in Korean]," *The Journal of Korean Acupuncture & Moxibustion Society*, vol. 24, no. 1, pp. 13-28, 2007.

Randomized Clinical Trials on Acupuncture in Korean Literature:

Bibliometric Analysis and Methodological Quality

No	Included article
69	W. S. Jung, C. M. Choi, J. W. Hong, et al., "The Clinical Effect of Manipulation of Acupuncture to Shen-Men and Nei-Kuan on Autonomic Nervous Function of Healthy Subjects [in English]," *Korean Journal of Oriental Medicine*, vol. 28, no. 4, pp. 69-73,

2007.

70 H. K. Kim, S. H. Lee, and Y. S. Kim, "Effects of Sa-am Acupuncture (Damjeonggyeok) on Autonomic Nervous Systerm in Night Nurses [in Korean]," *The Journal of Korean Acupuncture & Moxibustion Society*, vol. 24, no. 4, pp. 13-23, 2007.

71 J. E. Kim and K. M. Park, "The Differential Effects of Acupuncture on Postures of Healthy Subjects [in Korean]," *Journal of Biomedical Engineering Research*, vol. 28, no. 1, pp. 46-54, 2007.

72 S. W. Lee, J. M. Yun, J. W. Son, et al., "The Effect of Electroacupuncture on Upper-Extremity Spasticity of Stroke Patients [in Korean]," *Journal of Korean Oriental Internal Medicine*, vol. 28, no. 3, pp. 492-501, 2007.

73 Y. S. Kim, J. W. Hong, W. S. Jung, et al., "A Comparative Study of Motor Recovery form Stroke between High and Low Frequency Electrical Acupoint Stimulation [in Korean]," *Journal of Korean Oriental Medical Society*, vol. 28, no. 3, pp. 289-298, 2007.

74 H. B. Kim, M. H. Lee, S. Y. Lee, et al., "The Comparative Study on the Effect of Constitution-dependent Acupuncture Treatment for Idiopathic Parkinson's Disease on Heart Rate Variability [in Korean]," *The Journal of Korean Acupuncture & Moxibustion Society*, vol. 24, no. 3, pp. 163-174, 2007.

75 Y. C. Park, D. I. Chang, Y. H. Lee, and D. S. Park, "The Study on the Effect of Acupuncture Treatment in Patients with Idiopathic Parkinson's Disease [in Korean]," *The Journal of Korean Acupuncture & Moxibustion Society*, vol. 24, no. 4, pp. 43-54, 2007.

76 J. H. Cho, "A Pilot Study of the Difference between Gyejigongyeong-hwan and Gyejibongnyeong-hwan Combined Acupuncture Therapy on the Primary Dysmenorrhea [in Korean]," *Journal of Korean Oriental Internal Medicine*, vol. 20, no. 1, pp. 161-168, 2007.

77 I. S. Lee, H. M. Youn, K. K. Jung, et al., "Effect of Sa-am Acupuncture Treatment on the Dysmenorrhea (Pillot Study, Single Blind, Randomized, Sham Acupuncture, Controlled Clinical Trial) [in Korean]," *Journal of Meridian & Acupoint*, vol. 24, no. 3, pp. 63-79, 2007.

78 S. K. Lee, S. W. Lee, and D. H. Choi, "Effect of Acupuncture on P6 for Preventing Opioid-induced Nasea and Vomiting [in Korean]," *Korean Journal of Oriental Physiology & Pathology*, vol. 21, no. 6, pp. 1637-1640, 2007.

79 M. S. Song, C. H. Choi, D. H. Youn, J. H. Kim, and C. S. Na, "The effect of acupuncture using tonification and sedation manipulation at LI4(Hegu) and KI7(Fuliu) on the control of sweating [in Korean]," *Journal of Meridian & Acupoint*, vol. 25, no. 4, pp. 133-145, 2008.

80 J. H. Park, Y. B. Chae, H. J. Park and J. J. Lee, "A Visual Factor of Blinding of the Non-Penetrating Placebo Needle [in Korean]," *Journal of Meridian & Acupoint*, vol. 15, no. 4, pp. 175-185, 2008.

81 D. S. Oh, S. Y. Jung, A. R. Kim, et al., "A Crossover Clinical Trial to Determine the Effect of Siguan(Four Gates) Points on Gastrointestinal Motility Suppressed by Loperamide Administration [in Korean]," *Journal of Korean Oriental Medical Society,* vol. 29, no. 1, pp. 01-06, 2008.

82 I. C. Jung, S. R. Lee, Y. C. Park, et al., "The Effect of Sa-am Acupuncture Simjeongkyeok Treatment for Major Symptom of Hwa-byung [in Korean]," *Journal of Oriental Neuropsychiatry,* vol. 19, no. 1, pp. 01-18, 2008.

83 H. Y. Kwon and J. H. Kim, "The effects of Yanggnungchon(G34) acupuncture on the muscle fatigue [in Korean]," *Journal of Meridian & Acupoint,* vol. 25, no. 2, pp. 115-123, 2008.

84 I. S. Park, C. Y. Jung, M. K. Jang, et al., "A Randomized Clinical Trial of Local Acupoints Compared with Distal Acupoints in Degenerative Osteoarthritis on Knee [in Korean]," *The Journal of Korean Acupuncture & Moxibustion Society,* vol. 25, no. 2, pp. 227-242, 2008.

85 W. K. Min, Jang, "A Comparative study of Warm needling and Acupuncture on Osteoarthritis of the Knee - a Randomized Controlled Trial - [in English]," *PhD thesis : Kyunghee University,* 2008.

Randomized Clinical Trials on Acupuncture in Korean Literature:

Bibliometric Analysis and Methodological Quality

No	Included article
86	H. K. Jung, Y. P. Jeong, S. Y. Chiang, et al., "The Comparative Study on the Acupuncture at Affected Limb and Unaffected Limb on Treating Ankle Sprain of Acute Stage [in Korean]," *The Journal of Korean Acupuncture & Moxibustion Society,* vol. 25, no. 3, pp. 107-113, 2008.
87	J. M. Park, J. Y. Gwak, S. Y. Cho, et al., "Effects of Head Acupuncture Versus Upper and Lower Limgs Acupuncture on Singnal Activation of Blood Oxygen Level Dependent (BOLD) fMRI on the Brain and Somatosensory Cortex [in Korean]," *The Journal of Korean Acupuncture & Moxibustion Society,* vol. 25, no. 5, pp. 151-165, 2008.
88	B. M. Kwak, M. J. Kim, Y. M. Kim, et al., "Persisting Effects of Acupuncture Method for Chronic Tension-type Headache ; A Randomized Controlled Trial [in Korean]," *The Journal of Korean Acupuncture & Moxibustion Society,* vol. 25, no. 2, pp. 165-177, 2008.

89 D. S. Oh, J. Lee, J. Y. Kim, and S. M. Choi, "Nonlinear Aanlysis of Cardiotonic Effect of Acupuncture Treatment on Heart Rate Variability Assessed by 24-hour Holter Monitoring [in Korean]," *Korean Journal of Oriental Medicine,* vol. 14, no. 1, pp. 85-89, 2008.

90 H. S. Jeong, C. S. Yang, J. S. Nam, I. S. Jang, L. H. Kim, and E. S. Seo, "Short Time Effect of Caffeine on Heart Rate Variability and the Effect of Acupuncture at Neiguan (PC6): A Randomized Double Blind Pilot Study [in Korean]," *Journal of Korean Oriental Internal Medicine,* vol. 29, no. 3, pp. 778-786, 2008

91 S. U. Park, W. S. Jung, S. K. Moon, et al., "Effects of Acupuncture on Autonomic Nervous System in Normal Subject under Mental Stress [in Korean]," *Journal of Korean Oriental Medical Society,* vol. 29, no. 2, pp. 107-115, 2008

92 W. J. Choi, "A Comparative Study of the Differences among PC9, TE3, PC5 and TE1 and Their Effects on the Human Body [in Korean]," *PhD thesis : Sangji University,* 2008

93 J. Y. Kim, S. M. Jeong, C. K. Park, E. K. Min, and T. C. Wang, "The Clinical Effectiveness of Acupuncture at Palsa (BaXie) for Hand Fuction in Hemiparetic Patients after Stroke [in Korean]," *The Journal of Korean Acupuncture & Moxibustion,* vol. 25, no. 5, pp. 97-104, 2008.

94 H. S. Bae, A. S. Shin, S. U. Park, et al., " Effects of Acupuncture at ST36 on Blood Pressure and Endothelial Dependent Vasodilation in Hypertensive Patient [in Korean]," *Journal of Korean Oriental Internal Medicine,* vol. 29, no. 3, pp. 657-665, 2008.

95 D. H. Oh, "The Pilot Study on the Effect of SAAM Acupunture Treatment for Patients with Cancer-related Fatigue Randomized, a Single-blind, Controlled Trial [in Korean]," *Master's thesis: Sangji University,* 2009.

96 B. K. Son, "Efficacy of the A-Shi Point Acupuncture on Acne Vulgaris : A Randomized, Double Blinded, Controlled Clinical Trial [in Korean]," *Master's thesis: Kyunghee University,* 2009.

97 J. H. Park, "The Neural Substrates of Verum Acupuncture Compared to Non-penetrating Placebo Needle: an fMRI Study [in Korean]," *PhD thesis: Kyunghee University,* 2009.

98 Y. Y. Chae, J. C. Lee, K. M. Park, et al., "Inhibitory Effect of Acupuncture at HT7 on the Sympathetic Activations to Smoking-related Visual Cues During Smoking Cssation [in Korean]," *Journal of Meridian & Acupoint,* vol. 26, no. 4, pp. 39-52, 2009.

99 M. S. Jeong, W. J. Choi, K. W. Lee, et al., "The Effects of Acupuncture Stimulation on Skin Conductance Response of Anxiety Patients and Normal Subjects [in Korean]," *Journal of Oriental Neuropsychiatry,* vol. 20, no. 2, pp. 101-110, 2009.

100 M. H. Min, Y. G. Choi, Y. J. Kim, et al., "The Effects of Sa-am acupuncture on Knee

Osteoarthritis [in English]," *Journal of Meridian & Acupoint,* vol. 26, no. 4, pp. 55-66, 2009

101 M. S. Kang and L. H. Kim, " The Effects of Mental Stress Stimulation and Acupuncture at Shinmun(HT7) on Heart Rate Variability [in Korean]," *Journal of Oriental Neuropsychiatry,* vol. 20, no. 1, pp. 165-176, 2009.

102 M. S. Han, " Effect of 5 Element Acupuncture Treatment on the Primary Dysmenorrhea [in Korean]," *Master's thesis: Dong Eui University,* 2009.

Randomized Clinical Trials on Acupuncture in Korean Literature:

Bibliometric Analysis and Methodological Quality

No	Included article
103	J. Y. Chung, J. I. Kim, S. H. Lee, and S. K. Kang, "Effects of Electro acupuncture on Parameters Related to Obesity in Adults with Abdominal Obesity: Three arm Randomized Single Blind Pilot [in Korean]," *The Journal of Korean Acupuncture & Moxibustion,* vol. 27, no. 6, pp. 43-57, 2010.
104	H. J. Kwon, J. K. Kim, S. H. Lee, C. H. Kim, and Y. S. Kim, "Effects of Sim-eui Point on Allergic Rhinitis Rhinosinusitis, and Other Causes of Nasal [in English]," *The Journal of Korean Acupuncture & Moxibustion,* vol. 27, no. 3, pp. 127-135, 2010.
105	J. Y. Park, M. S. Kim, J. C. Jeon, et al, "Comparative Study of Sosang(LU11)-Eunbaek(SP1) Bloodletting and Sa-Kwan(LI4 and LR3) Acupuncture on Acute [in Korean]," *The Journal of Korean Acupuncture & Moxibustion,* vol. 27, no. 1, pp. 129-135, 2010.
106	S. W. Kim, "Efficct of Acupuncture at Shinmun(HT7) on EEG and HRV in Different Stress response Levels [in Korean]," *Master's thesis: Kyunghee University,* 2010.
107	S. J. Kim, H. Lee, H. S. Jung, et al., "A Clinical Study on Effect of Electro-acupuncture Treatment for Lumbago Patients Caused by Traffic [in Korean]," *The Journal of Korean Acupuncture & Moxibustion,* vol. 27, no. 5, pp. 117-123, 2010.
108	K. S. Yoon, H. Lee, J. H. Kang, and J. Y. Choi, "Comparison Study on 30 Cases of HIVD Patients with Restricted on SLRT by Sa-Am Acupuncture Banggwangjeonggyeok and General Acupuncture [in Korean]," *The Journal of Korean Acupuncture & Moxibustion,* vol. 27, no. 5, pp. 79-87, 2010.
109	C. H. Woo, O. G. Kwon, and H. D. An, hoi, "The Comparative Study on the Effect of Acupuncture Treatment with or without Indirect Moxibustion Treatment for Acute Ankle Sprain Comparison [in Korean]," *Journal of Oriental Rehabilitation Medicine,* vol. 20, no. 1, pp. 141-152, 2010.

110 S. H. Kim, J. S. Kim, B. H. Lee, S. C. Lim, T. Y. Jung, and K. M. Lee, "Comparative Clinical Study of Jung-an Acupuncture and General Acupuncture on Bell′s Palsy Patients[in Korean]," *The Journal of Korean Acupuncture & Moxibustion,* vol. 27, no. 1, pp. 43-49, 2010.

111 Y. J. Choi, K. J. Yoon, M. S. Kim, et al., "Effects of Scalp Acupuncture with Usual Acupuncture on Peripheral Facial Palsy in Comparison with Usual Acupuncture Only [in Korean]," *The Journal of Korean Acupuncture & Moxibustion,* vol. 27, no. 6, pp. 101-109, 2010.

112 J. H. Lee, Y. J. Choi, B. C. Shin, and S. T. Koo, " Differential Effects of Two Individual Acupuncture Points (BL62, KI6) on Heart Rate Variability in Healthy Volunteers : A Randomized, Single-Blind, Self-Controlled [in Korean]," *Journal of Meridian & Acupoint,* vol. 27, no. 4, pp. 85-96, 2010.

113 K. H. Kim, D. I. Kim, D. S. Hwang, et al., "Methodologic Lessons Learned from Multicenter, Randomized, Controlled Clinical Trial of Acupuncture for Hot Flashes in Peri- and Postmenopausal Women [in Korean]," *The Journal of Oriental Obstetrics & Gynecology,* vol. 23, no. 1, pp. 042-052, 2010.

114 S. W. Jeon, "The Effect of Acupuncture on Tinnitus: A Randomized Controlled Clinical Trial [in Korean]," *Master′s thesis: Kyunghee University,* 2011.

115 K. S. Kim, "Anti-inflammatory effect of Keigai-rengyo-to extract and acupuncture in male patients with acne vulgaris: A randomized controlled [in English]," *PhD thesis: Kyunghee University,* 2011.

116 W. J. Choi, S. G. Lee, I. B. Son, and S. H. Sun, "The effects of Sa-am Acupuncture Simpojeongkyeok Treatment on Hwa-byung : Randomized, patient-assessor blind, placebo-controlled acupuncture, pilot clinical [in Korean]" *The Journal of Korean Oriental Medical Society,* vol. 22, no. 2, pp. 1-13, 2011.

117 H. Y. Kwak, "Acupuncture for Whiplash injury - A feasibility pilot study- [in Korean]," *Master′s thesis: Kyunghee University,* 2011.

118 J. Y. Park, K. J. Yun, Y. J. Choi, et al., "Comparative Study of Treatment Effect between Near Acupuncture Point Needling and Near Acupuncture with Remote Acupuncture Point Needling on Treatment of Posterior Neck Pain [in Korean]," *The Journal of Korean Acupuncture & Moxibustion,* vol. 28, no. 1, pp. 85-92, 2011.

Randomized Clinical Trials on Acupuncture in Korean Literature:

Bibliometric Analysis and Methodological Quality

No	Included article
119	J. B. Lee, J. G. Im, H. G. Lee, J. U. Kim, T. H. Yook, and B. Y. Song, "The Comparison of Effectiveness between Acupuncture and Its Cotreatment with Wan-Gwa Acupuncture

on the Treatment of Low Back Pain [in Korean]," *The Journal of Korean Acupuncture & Moxibustion,* vol. 28, no. 2, pp. 43-47, 2011.

120 J. H. Yim, S. H. Yoon, W. K. Jung, H. S. Sin, S. H. Cho, and S. K. Kim, "With or without Acupuncture Treatment on Peroneus Muscles for the Treatment in Patients with Acute Inversion Sprain of Ankle : A Comparative Trial [in Korean]," *Journal of Oriental Rehabilitation Medicine,* vol. 21, no. 2, pp. 289-297, 2011.

121 J. H. Yim, S. H. Yoon, W. S. Jung, H. S. Sin, S. H. Cho, "The Comparative Study of Improvement of Patient Who were Diagnosed Sprain and Strain of Lumbar Sine with MET Treatment of Iliosoas Muscles and with Acupuncture Treatment [in Korean]," *The Journal of Korea CHUNA Manual Medicine for Spine & Nerves,* vol. 6, no. 1, pp. 1-9, 2011.

122 D. H. Chang, U. Y. Bae, J. H. Jung, and I. S. Lee, "The Effects of Burning Acupuncture Theraphy with Chuna Therapy for Low Back Pain Patients [in Korean]," *Journal of Oriental Rehabilitation Medicine,* vol. 21, no. 3, pp. 21-32, 2011.

123 J. B. Lee, J. G. Im, H. G. Lee, T. H. Yook, and J. U. Kim, "Comparison of Effectiveness between Acupuncture and its Cotreatment with Foot Acupuncture on Low Back Pain [in Korean]," *The Journal of Korean Acupuncture & Moxibustion,* vol. 28, no. 4, pp. 1-7, 2011.

124 S. W. Park, "A Randomized Controlled Trial of Acupuncture treatment on Cervicalgia [in Korean]," *Master's thesis : WonKang University,* 2011.

125 S. Y. Han, j. Y. Lee, S. H. Park, et al., "A Clinical Study on Effect of Electro-acupuncture Treatment for Whiplash Injury Patients Caused by Traffic Accident [in Korean]," *The Journal of Korean Acupuncture & Moxibustion,* vol. 28, no. 6, pp. 107-115, 2011.

126 Y. K. Jo, S. S. Kim, J. S. Lee, and S. H. Chung, "Comparison of the Effect of Superficial Acupuncture and Deep Acupuncture on Finger Extensor Muscle Function of Normal Person : Randomized Controlled Trial [in Korean]," *The Journal of Korea CHUNA Manual Medicine for Spine & Nerves,* vol. 6, no. 1, pp. 97-104, 2011.

127 S. K. Lim, D. H. Lee, Y. J. Kwon, et al., "Effects of Fixed-intensity and Varied-intensity Electroacupuncture on Heart Rate Variability in Healthy Peope with Stress Task [in Korean]," *The Journal of Korean Acupuncture & Moxibustion,* vol. 28, no. 2, pp. 107-116, 2011.

128 J. S. Park, M. S. Ahn, J. J. Lee, et al., "Study on the Effects of Acupuncture at Jeonjung(CV17) on the Heart Rate Variability in Healthy Adults [in Korean]," *The Journal of Korean Acupuncture & Moxibustion,* vol. 28, no. 2, pp. 13-25, 2011

129 Y. J. Yun, K. W. Kang, J. N. Yang, M. S. Chun, J. B. Choi, and J. I. Yang, "Effects of Acupuncture on Urinary Incontinence in Premenopausal Women : Preliminary Study [in Korean]," *The Journal of Korean Acupuncture & Moxibustion,* vol. 28, no. 3, pp.

55-71, 2011

130 G. E. Lee, N. K. Kim, H. Y. Kim, and H. W. Kang, "The Effects of Acupuncture Treatment on Hwa-byung patient's Insomnia: Patient-assessor blind, Randomized, Placebo-controlled Clinical trial [in Korean]," *Journal of Oriental Neuropsychiatry,* vol. 23, no. 1, pp. 31-48, 2012

131 M. J. Oh and H. S. Song, "Effect of Sa-Am Acupuncture Bladder Reinforcing Method to Ryodoraku on the Patients with Chronic Low Back Pain [in Korean]," *The Journal of Korean Acupuncture & Moxibustion,* vol. 29, no. 2, pp. 37-42, 2012

132 M. J. Oh, H. S. Song, "Effect of Acupuncture Treatment on Ryodoraku Score of the Patients with Chronic Low Back Pain Due to the Kidney Deficiency [in Korean]," *The Journal of Korean Acupuncture & Moxibustion,* vol. 29, no. 3, pp. 115-120, 2012

133 S. M. Lee and J. S. Lee, " The Comparison of Superficial and Deep Acupuncture on the Ashi-point of Elbow Pain Patients Effect [in Korean]," *Journal of Oriental Rehabilitation Medicine,* vol. 22, no. 3, pp. 193-203, 2012.

134 C. H. Yeon and S. H Chung, "Muscle Belly and Acupuncture Treatment at Myotendinal Junction on Delayed-Onset Muscle Soreness [in Korean]," *Journal of Oriental Rehabilitation Medicine,* vol. 22, no. 2, pp. 219-223, 2012.

135 H. M. Kim, "(The) Efficacy of Acupuncture on the Circadian Rhythm of Blood Pressure : A Randomized, Double-Blinded, Controlled Trial [in English]," *Master's thesis : Kyunghee University,* 2012.

Randomized Clinical Trials on Acupuncture in Korean Literature:

Bibliometric Analysis and Methodological Quality

No	Included article
136	J. H. Yoo, K. W. Kim, D. H. Kim, et al., "A Controlled Trial on the Effect of Complex Oriental Medical Treatment with or without Pyung-Hyung Acupuncture on the Treatment of Peripheral Facial Palsy [in Korean]," *The Journal of Korean Acupuncture & Moxibustion,* vol. 29, no. 3, pp. 121-128, 2012
137	J. N. Kim, J. S. Lee, J. S. Hong, S. J. Kim, and S. I. Moon, "Effect of Simple Acupuncture and Electroacupuncture at Bai Hui(GV20) on Heart Rate Variability in Healthy Adults with Stress Task [in Korean]," *The Journal of Korean Acupuncture & Moxibustion,* vol. 29, no. 3, pp. 89-99, 2012
138	N. S. Kim, S. J. Kim, H. J. Ryu, S. S. Nam, and Y. S. Kim, "Effects of Taegeuk Acupuncture on Autonomic Nervous System by Analyzing Heart Rat Variability in Soyangin [in Korean]," *The Journal of Korean Acupuncture & Moxibustion,* vol. 29, no. 3, pp. 81-88, 2012

[139] Y. J. Kwon, D.H Lee, U. I. Lee, K. M. Park and S. H. Lee, "Effects of Fixed-intensity and Varied-intensity Electroacupuncture in Pain and Sensory Threshold in Patients with Chronic Tension Headache [in Korean]," *The Journal of Korean Acupuncture & Moxibustion*, vol. 29, no. 4, pp. 25-34, 2012

[140] J. H. Jo, J. J. Kweon, Y. K. Song, H. H. Lim, H. J. Beak, H, "Acupuncture's Efficacy and Safety in Axial Spondyloarthritis within 4 Weeks Session : a Randomized, Double-blind, Sham-controlled Trial [in Korean]," *Journal of Oriental Rehabilitation Medicine*, vol. 22, no. 4, pp. 23-36, 2012

[141] W. Lie, N. R. Lee, W. H. Choi, S. B. Kim, Y. H. Lee, T. M. Shin, "Analysis of Pupil Size Variability for Effectiveness Verification of Activation Level of Autonomic Nerve System by Electromagnetic Acupuncture System [in Korean]]," *Journal of Meridian & Acupoint*, vol. 29, no. 4, pp. 554-562, 2012.

[142] Y. S. Hwang, C. S. Park, S. T. Koo, "Effect of acupuncture on the Muscle Fatigue Recovery in Different Diameters of Needle [in Korean]," *Journal of Meridian & Acupoint*, vol. 29, no. 4, pp. 364-642, 2012.

[143] J. E. Kim, K. W. Kang, A. R. Kim, et al., "Acupuncture for Chronic Fatigue Syndrome and Idiopathic Chronic Fatigue: a Pilot Randomized Controlled Trial, [in Korean]," *The Journal of Korean Acupuncture & Moxibustion*, vol. 29, no. 5, pp. 109-118, 2012

[144] *W. K. Min, S.J. Yeo, E. H. Kim, et al“Comparison of arm"""Needling and Acupuncture for Knee Osteoarthritis: A Randomized Controlled Trial[in English],”Korean journal of acupunture, vol.30, no. 1, pp64-72, 2013.*

	**Randomized Clinical Trials on Pharmaco Acupuncture in Korean Literature: Bibliometric Analysis and Methodological Quality (N=70)**
**No**	**Included article**
1	H. S. Yoo et al., "The Effect of Sweet Bee Venom Pharmacouncture(SBVP) on Cancer-Related Pain : A Randomized Controlled Trial and Double Blinded – Pilot study [in English]," *Journal of Pharmacopuncture*, vol. 11, no. 1, pp. 21-29, 2008.,
2	J. H. Kim et al., "The Effect of Mahuang(Ephedra sinica Stapf) – Chuanwu(Aconitum carmichaeli Debx) Pharmacopuncture Treatment in Obese Patients [in English]," *The Journal of Korean Acupuncture & Moxibustion Society*, vol. 26, no. 5, pp. 77-83, 2009.,
3	A. R. Lee et al., "The Effect of Sobi-eum(Xiaofei-yin) Mesotherapy on Abdominal Fat Distribution [in English]," *Journal of Oriental Rehabilitation Medicine*, vol. 19, no. 2, pp. 261-273, 2009.
4	J. H. Park et al., "The Efficacy and Safety of Ephedra and Green Tea Mesotherapy on Localized Fat [in English]," *Journal of Society of Korean Medicine for Obesity Research*, vol. 7, no. 1, pp. 71-85, 2007.
5	C. S. Lim et al., "Clinical Studies of Sweet Bee Venom to the Effect of Abdominal Fat Accumulation [in English]," *Journal of Pharmacopuncture*, vol. 11, no. 2, pp. 55-62, 2008.
6	J. S. Lee et al., "A Study on Allergic responses Between Bee Venom and Sweet Bee Venom Pharmacopuncture [in English]," *Journal of Pharmacopuncture*, vol. 9, no. 3, pp. 61-77, 2006.
7	K. R. Kwon, "A Clinical Study on the Effects of Intravenous Wild ginseng Herbal Acupuncture on the Human Body [in English]," *Journal of Pharmacopuncture*, vol. 7, no. 1, pp. 15-26, 2004.
8	J. W. Park et al., "A Clinical Pilot Study of Carthami-Semen Herbal Acupuncture Treatment for Chronic Constipation [in English]," *The Journal of Korean Acupuncture & Moxibustion Society*, vol. 25, no. 5, pp. 127-137, 2008.,
9	S. H. Lee et al., "Randomized Controlled Double Blind Study of Bee Venom Therapy on Rheumatoid Arthritis [in English]," *The Journal of Korean Acupuncture & Moxibustion Society*, vol. 20, no. 6, pp. 80-88, 2003.,
10	B. Y. Song, "A Clinical Study on the Effects of Sweet Bee Venom Herbal Acupuncture for Patients with Whiplash Injury [in English]," *Journal of Pharmacopuncture*, vol. 10,

no. 3, pp. 77-83, 2007.,

11 S. Hyun et al., "Effects of Jungsongouhyul Herbal Acupuncture(JSO) Multi-treatment for Whiplash Injury by Traffic Accident [in English]," *Journal of Pharmacopuncture*, vol. 8, no. 1, pp. 59-65, 2005.,

12 K. H. Lee et al., "Comparison of Treatment Effects and Allergic responses to stiff neck between Sweet Bee Venom and Bee Venom Pharmacopuncture [in English]," *Journal of Pharmacopuncture*, vol. 11, no. 4, pp. 39-48, 2008.,

13 K. T. Kim et al., "A Randomized Controlled Double Blinding Study of Bee Venom Acupuncture Therapy on Sprain of C-spine [in English]," *The Journal of Korean Acupuncture & Moxibustion Society*, vol. 22, no. 4, pp. 189-195, 2005.,

14 Y. H. Kang et al., "The Clinical Effects of Korean Bee-Venom Therapy in Neck Pain Due tot Soft Tissue Damage [in English]," *The Journal of Korean Acupuncture & Moxibustion Society*, vol. 19, no. 6, pp. 67-79, 2002.,

15 H. E. Kim et al., "The Clinical Effects of Carthami-Flos Herbal Acupuncture in Neck Pain Due to Soft Tissue Damage [in English]," *The Journal of Korean Acupuncture & Moxibustion Society*, vol. 19, no. 4, pp. 112-123, 2002.,

16 W. M. Na et al., "A Study on Pain relief effects and Allergic resonses for the Osteoarthritis of the knee joint Between Sweet Bee Venom and Bee Venom Pharmacopuncture [in English]," *Journal of Pharmacopuncture*, vol. 10, no. 2, pp. 47-55, 2007.,

17 S. M. Ryu et al., "The Effect of Intra-articular Bee Venom Injection on Osteoarthritis of the Knee [in English]," *The Journal of Oriental Rehabilitation Medicine*, vol. 14, no. 1, pp. 35-52, 2004.,

18 S. N. Lee et al., "The Clinical Study on Bee Venom Acupuncture Treatment on Osteoarthritis of Knee Joint [in English]," *The Journal of Korean Acupuncture & Moxibustion Society*, vol. 20, no. 5, pp. 73-81, 2003.,

19 K. B. Park et al., "Study on Clinical Effects of Homnis Placenta Herbal Acupuncture on Osteoarthritis of Knee Joint [in English]," *The Journal of Korean Acupuncture & Moxibustion Society*, vol. 23, no. 4, pp. 163-173, 2006.,

20 H. B. Kim et al., "Comparative Study of Effects of ʻIntramuscular Bee Venom Herbal Acupunctureʼ and ʻIntracutaneous Bee Venom Herbal Acupunctureʼ in Knee Osteoarthritis Patients [in English]," *The Journal of Korean Acupuncture & Moxibustion Society*, vol. 25, no. 2, pp. 151-164, 2008.,

21 K. R. Yang et al., "A Comparative study of Warm needling and Bee Venom Pharmacopuncture on Osteoarthritis of the Knee – a Randomized Controlled Trial [in English]," *Journal of Pharmacopuncture*, vol. 11, no. 2, pp. 21-31, 2008.,

22 B. J. An, K. T. Kim, M. S. Kang and H. S. Song, "Effect of Bee Venom-acupuncture on Patients with Osteoarthritis of Knee Joint [in Korean]," *The Journal of Korean Acupuncture & Moxibustion Society*, vol. 23, no. 4, pp. 15-20, 2006

23 B. J. An and H. S. Song, "Effect of Bee Venom-acupuncture on Patients with Sprain of the Wrist Joint [in Korean]," *Journal of Pharmacopuncture*, vol. 9, no. 2, pp. 167-171, 2006

24 J. Y. Ku, K. H. Lee, S. W. Cho, S. C. Lee, H. M. Youn, K. J. Jang, C. H. Song, C. B. Ahn and C. H. Kim, "Comparison of the Effects between Sweet Bee Venom Pharmacopuncture and Scolopendrid Pharmacopuncture on Carpal Tunnel Syndrome (Randomized, Controlled Clinical Trial) [in Korean]," *Journal of Pharmacopuncture*, vol. 13, no. 4, pp. 75-89, 2010

25 J. A. Lim, S. C. Kim, S. N. Kim, S. Y. Lee and H. C. Moon, "Clinical study on treatment of Carpal tunnel syndrome using Scolopendrid herbal acupuncture [in Korean]," *Journal of Pharmacopuncture*, vol. 8, no. 1, pp. 13-20, 2005

26 J. H. Kim, S. H. Jang, H. M. Yoon, K. J. Jang, C. B. Ahn, C. H. Kim, C. H. Song and H. N. Choi, "The comparison of Effectiveness between Bee Venom and Sweet Bee Venom Therapy on Chronic Lower Back Pain [in Korean]," *Journal of Pharmacopuncture*, vol. 11, no. 4, pp. 15-24, 2008

27 T. H. Lee, H. S. Hwang, S. Y. Chang, J. H. Cha, K. H. Jung, E. Y. Lee and J. D. Roh, " The Comparison of Effectiveness between Bee Venom and Sweet Bee Venom Therapy on Low back pain with Radiating pain [in Korean]," *Journal of Pharmacopuncture*, vol. 10, no. 3, pp. 85-89, 2007

28 S. H. Lee, M. W. Kang, H. Lee and S. Y. Lee, "Effectiveness of Bee-Venom acupuncture on Ouhyul Herbal Acupuncture in Herniation of Nucleus Pulposus-comparison with Acupuncture Therapy Only [in Korean]," *The Journal of Korean Acupuncture & Moxibustion Society*, vol. 24, no. 5, pp. 197-205, 2007

29 S. M. Yu, J. Y. Lee, K. R. Kwon and H. S. Lee, "Comparative Study of Acupuncture, Bee Venom Acupuncture, and Bee Venom Pharmacopuncture on the Treatment of Herniation of Nucleus Pulpous [in Korean]," *The Journal of Korean Acupuncture & Moxibustion Society*, vol. 23, no. 5, pp. 39-54, 2006

30 W. S. Chung, J. S. Lee, S. H. Chung and S. S. Kim, "The Effect of Bee Venom Acupuncture on Patient with Herniation of Nucleus Pulposus of Lumbar Spine [in Korean]," *Journal of Oriental Rehabilitation Medicine*, vol. 13, no. 2, pp. 87-101, 2003

31 K. T. Kim and H. S. Song, "The Effectiveness of Bee Venom Acupuncture Therapy on the Treatment of Sprain of L-spine(A Randomized Controlled Trial; Double Blinding) [in Korean]," *The Journal of Korean Acupuncture & Moxibustion Society*, vol. 22, no. 4, pp. 113-120, 2005

32 H. Lee, "The Comparative Study on the Bee-Venom Therapy and Common Acupuncture Therapy for the Acute Ankle Sprain [in Korean]," *The Korean Journal of M eridian & A cupoint*, vol. 21, no. 4, pp. 133-143, 2004

33 H. S. Song, "The Comparative Study on the Bee-Venom Therapy and Common Acupuncture Therapy for the Acute Ankle Sprain [in Korean]," *The Korean Journal of M eridian & A cupoint*, vol. 21, no. 4, pp. 133-143, 2004

34 I. Kang, J. Y. Moon and M. J. Lim, "The Comparison Study between Different Interventions for Treating Acute Ankle Sprain - Using Dry Needle, Bee Venom Acupuncture, Hwangryunhaedoktang Herbal Acupuncture - [in Korean]," *The Journal of Korean Acupuncture & Moxibustion Society*, vol. 25, no. 5, pp. 1-7, 2008

35 J. W. Seo, M. J. Park, I. H. Sung, N. O. Kim and C. K. Ahn, "A Clinical Study of Bee Venom Acupuncture Therapy on the Treatment of Acute Ankle Sprain [in Korean]," *The Journal of Korean Acupuncture & Moxibustion Society*, vol. 23, no. 1, pp. 95-103, 2006

36 G. H. An, H. Lee and B. R. Lee, "The Comparative Study on the Bee-Venom Therapy and Common Acupuncture Therapy for the Lateral Epicondylitis (Tennis Elbow) [in Korean]," *Journal of Taejon University*, vol. 13, pp. 267-276, 2004

37 S. Y. Jeong, Z. W. Park, J. M. Shin, J. Y. Kim and I. Y. Youn, "The Comparative Study of Effectiveness between Acupuncture and its Cotreatment with CalCulus Bovis · Fel Ursi · Moschus Parmacopuncture on the Treatment of Acute Low Back Pain [in Korean]," *The Journal of Korean Acupuncture & Moxibustion Society*, vol. 28, no. 4, pp. 105-110, 2011

38 H. Y. Shin, S. M. Lee, J. H. Kim, S. J. Kim, Y. J. Choi, T. Y. Jung, J. S. Kim, S. C. Lim, Y. K. Lee, B. H. Lee and K. M. Lee, "Comparative Study of Effects on Intracutaneous Bee Venom Pharmacopuncture and Intramuscular Bee Venom Pharmacopuncture in Lumbar Disc Herniation [in Korean]," *The Journal of Korean Acupuncture & Moxibustion Society*, vol. 28, no. 3, pp. 1-11, 2011

39 Y. J. Shin, "A Clinical Pilot Study Comparing Sweet Bee Venom parallel treatment with only Acupuncture Treatment in patient diagnosed with lumbar spine sprain [in Korean]," *Journal of Pharmacopuncture*, vol. 14, no. 2, pp. 37-43, 2011

40 B. C. Jun, E. S. Kim, D. S. Kim, T. H. Kim, J. Y. Kim, "Effeciveness of ShinBaro Pharmacopuncture on Lumbar Spinal Herniated interverterbral Disc" [in Korean]," *The Journal of Korea CHUNA Manual Medicine for Spine & Nerves*, vol. 6, no. 2, pp. 109-119, 2011.

41 G. M. Lim, J. H. Kim, E. H. Hwang, T. H. Yook, Y. S. Ko, "The Effects of Bee Venom Pharmacopuncture on Muscle Activity and Pain Reception of Upper Limbs" [in Korean]," *The Journal of Oreiental Rehabilitation medicine*, vol. 21, no. 3, pp. 57-69, 2011.

42 J. H. Lee, J. S. Kim, K. Y. Yang, S. H. Han, C. R. Lee, H. K. Shin, "The Comparative Study on the Effect of Pharmacopuncture Treatment and Chuna Treatment for Neck Pain Caused by Traffic Accidents" [in Korean]," *The Journal of Oreiental Rehabilitation medicine*, vol. 22, no. 1, pp. 75-83, 2012.

43 J. Y. Park, K. J. Yun, Y. J. Choi, M. S. Kim, S. J. Yu, C. K. Lee, J. D. Roh, E. Y. Lee, "The Clinical Effects of Carthami-Flos Pharmacopuncture on Posterior Nect pain of Menopausal Women" [in Korean]," *Journal of Pharmacopuncture*, vol. 14, no. 4, pp. 71-80, 2011

44 J. G. Im, J. B. Lee, H. G. Lee, T. H. Yook, J. U. Kim, "Effects of the Acupuncture Therapy in Combination with Soyeom Pharmacopuncture Therapy on Acute Whiplash Injury by Traffic Accident" [in Korean]," *The Jounal of Korean Acupuncture & Moxibustion Society*, vol. 28, no. 4, pp. 9-18, 2011

45 P. S. Koh, "Effectiveness of Combined Bee Venom Acpuncture and Physiotherapy on Frozen Shoulder – a randomized controlled, patient-assessor blinded trial" [in English]," *Degree of Master of Science in Oriental Medicine – Kyung Hee University*.

46 J. Y. Choi, H. Lee, J. H. Kang, Y. I. Kim, J. H. Kim, S. H. Lee, N. Y. Kim, Y. K. Yim, "Comparative Study of General Oriental Medical Treatment and Bee Venom Pharmacopuncture on Acute Peripheral Facial Paralysis Patient with Postauricular Pain" [in Korean]," *The Jounal of Korean Acupuncture & Moxibustion Society*, vol. 26, no. 5, pp. 95-103, 2009

47 B. C. Choi, K. S. Han, T. W. Ahn, "Clinical comparison studies on 30 casies of Bell's palsy patient with postauricular pain by Anti-inflammatory pharmacopuncture & Acupuncture and Herbal therapy" [in Korean]," *Daejeon university*

48 H. K. Kim, "Clinical Studies on Hwangryunhaedoktang Herbal-acupuncture Therapy on functional Headache" [in Korean]," *Journal of Pharmacopuncture*, vol. 9, no. 3, pp. 131-138, 2006

49 M. S. Kim, H. J. Kim, Y. J. Park, E. H. Kim, E. Y. Lee, "The clinical research of the efficacy of bee venom aqua-acupuncture on peripheral facial paralysis" [in Korean]," *The Jounal of Korean Acupuncture & Moxibustion Society*, vol. 21, no. 4, pp. 251-262, 2004

50 H. Seol, T. H. Yook, " Effects of Hwangryunhaedoktang Herbal-acupuncture at G21(Kyonjong : 肩井) on the Heart Rate Variability" [in Korean]," *The Jounal of Korean Acupuncture & Moxibustion Society*, vol. 21, no. 6, pp. 37-50, 2004

51 H. W. Shin, J. H. Kang, H. Lee, "Efficacy of Soyeom Pharmacopuncture on Postauricular Pain Accompanied with Peripheral Facial Paralysis" [in Korean]," *The Jounal of Korean Acupuncture & Moxibustion Society*, vol. 25, no. 6, pp. 41-49, 2009

52 H. J. Kim, B. Y. Song, T. H. Wook, "The Effects of distilled Cervi Pantotrichum Cornu Pharmacopunture and Zizyphi Spinosi Semen Pharmacopunture on the Heart Rate Variability" [in Korean]," *Journal of Pharmacopuncture,* vol. 12, no. 3, pp. 31-40, 2009

53 J.D.R, L.H.K, B.Y.S, et al. "The Effects of distilled Wild Ginseng Herbal Acupuncture on the Heart Rate Variability(HRV)", [in Korean], *Journal of Pharmacopuncture,* vol.11, no.1, pp.55-69, 2008

54 J.C.S, L,H.K, B.Y.S, et al. "The Effects of distilled Rehmannia glutinosa Herbal Acupuncture on the Heart Rate Variability(HRV)", [in Korean], *Journal of, Pharmacopuncture*, vol.11, no.1, pp.83-97, 2008

55 H.S, B.Y.S, T.H.Y. "The Effects of Panax Ginseng Radix Pharmacopuncture and Zizyphi Spinosi Semen Pharmacopuncture on the Heart Rate Variability", [in Korean], *The Journal of Korean Acupuncture and Moxibustion society*, vol.26, no.5, pp.20-28, 2009

56 S.Y.C, S.Y.H, J.Y.J, et al. "Effect of Hwangryunhaedok-tang Pharmacopuncture at CV17(Jeonjung:膻中) for Mental Stress on Short-term Analysis of Heart Rate Variability", [in Korean], *The Journal of Korean Acupuncture and Moxibustion society*, vol.26, no.5, pp.49-56, 2009

57 J.B.L, B.Y.S, T.H.Y. "The Effects of Carthami Semen Pharmacopuncture and Bovis Calculus·Fei Ursi Pharmacopuncture on the Heart Rate Variability(HRV)", [in Korean], *The Journal of Korean Acupuncture and Moxibustion society*, vol.27, no.2, pp.11-21, 2010

58 S.T.L, L.H.K, B.Y.S, et al. "The Effects of distilled Astragali Radix Herbal Acupuncture on the Heart Rate Variablility(HRV), [in Korean], *Journal of Oriental Neuropsychiatry*, vol.19, no.1, pp.107-124, 2008

59 S.W.C, K.H.G, J.H.N, et al. "Ther Effectiveness of Zingiberis Rhizoma Herbal Acupuncture Therapy and Bee Venom Herbal Acupuncture Therapy on the Poststroke Hemiplegic Shoulder Pain" [in Korean], *Journal of Oriental Rehabilitation Medicine*, vol.15, no.4, pp.77-87, 2005

60 D.Y.L, G.M.L, S.C.Y, et al. "A clinical Study of Bee Venom Acupuncture Therapy on Shoulder Pain Patients in Stroke Sequelae", [in Korean], *The Journal of Korean Acupuncture and Moxibustion society*, vol.23, no.4, pp.69-80, 2006

61 Y.E.J, H.W.S, R.K.K, et al. "Comparative study of Acupuncture, Bee Venom Acupuncture and Bee Venom Herbal Acupuncture on the treatment of Post-stroke Hemiplegic Shoulder Pain", [in Korean], *Journal of Pharmacopuncture,* vol.9, no.1, pp.139-154, 2006

[62] C.N.K, I.K.M, S.W.P, et al. "Effectiveness of Bee Venom Acupuncture on Shoulder Pain after Stroke", [in Korean], *Journal of Korean Oriental Medical Society* , vol.28, no.1, pp.11-24, 2007

[63] H.H.B, Y.C.P. "Jungsongouhyul Pharmacopuncture simultaneous treatment stroke induced shoulder pain analysis of the effects of muscle strength exercise on physical function and quality of life, Randomized, Controlled Clinical Trial)", [in Korean], *Journal of Pharmacopuncture*, vol.7, no.1, pp.77-86, 2004

[64] J.H.N, J.A.P, S.W.C, et al. "Effect of Bee-venom Acupuncture on Upper Limb Spasticity of Stroke Patients", [in Korean], *The Journal of Korean Acupuncture and Moxibustion society*, vol.27, no.4, pp.115-125, 2010

[65] J.H.N, J.A.P, H.M.Y, et al. "The effect of Hominis Placenta Pharmacopuncture on Leg spasticity of stroke patients(A Pilot study, Double blind, Randomized, Controlled Clinical Trial)", [in Korean], *Journal of Pharmacopuncture*, vol.12, no.4, pp.97-110, 2009

[66] J.Y.G, S.Y.C, A.S.S, et al. "Efficacy of Bee-venom Acupuncture on Central Post Stroke Pain －Single-blind Randomized Controlled Trial－", [in Korean], *The Journal of Korean Acupuncture and Moxibustion society*, vol.26, no.6, pp.205-214, 2009

[67] J.A.P, C.H.L, G.S.K, et al. "The Effects of Sweet Bee Venom Pharmacopuncture on the Post-stroke Hemiplegic Shoulder Pain", [in Korean], *The Journal of Korean Acupuncture and Moxibustion society*, vol.28, no.4, pp.37-47, 2011

[68] C.H.L, J.Y.K, J.A.P, et al. "Comparison of the Cfficacy between Method of Regulating Ascending Kidney Water and Descending Heart Fire and Sweet Bee Venom Pharmacopuncture on Peripheral Facial Paralysis", [in Korean], *The Journal of Korean Acupuncture and Moxibustion society*, vol.28, no.4, pp.85-92, 2011

[69] T.H.K, K.Y.P, J.Y.P, et al. "The Effect of Hominis Placenta Herbal Aupuncture Therapy on the Postpartum Women's Heat Feeling, Sweat and Thirst" [in Korean], *The Journal of Oriental Obstetrics and Gynecology*, vol.23, no.3, pp.139-155, 2010

[70] S.M.K, S.H.J, C.H.K, et al. "Effect of Hominis placenta Pharmacopuncture on the Dysmenorrhea(A Pilot study, Single blind, Randomized, Controlled Clinical Trial)", [in Korean], *The Journal of Oriental Obstetrics and Gynecology*, vol.11, no.3, pp.123-131, 2008

Randomized Clinical Trials on Herbal Medicine in Korean Literature:

Bibliometric Analysis and Methodological Quality (N=30)

No	Included article
1	J.K.O, S.S.K, "Influence of Chungseoikgi-Tang on Recovery after Performing Exercise [in Korean]," *Journal of Oriental Rehabilitation Medicine*, vol.4, no.1, pp.101-109, 1994.
2	L.H.K, I.S.J, J.Y.K, et al, "A Clinical Trial about Anti-anxiety and Anti-stress Effect of a modified formula consisted with Several Herbs [in Korea]," *Korean Journal of Oriental Physiology & Pathology*, vol.17, no.6, pp.1533-1537, 2003.
3	S.J.K, H.J.K, B.P.K, et al, " Effect of Ephedra Sinica and Evodia Rutaecarpa on Resting Metabolic Rate in Obese Premenopausal Women during Low-calorie Diet : A Randomized Controlled Clinical Trial [in Korean]," *Journal of Korean Oriental Association for Study of Obesity*, vol.4, no.1, pp.45-54, 2004.
4	S.H.H, S.S.K, "Effect of Hwangryunhaedok-Tang Gamibang(Huanglianjiedu-Tang Jiaweifang) Cerebral Blood Flow in Subacute Stage Stroke Patients [in Korean]," *Journal of Oriental Rehabilitation Medicine*, vol.14, no.4, 2004.
5	I.S.Jang, C.N.Ko, I. L, et al, " Effect of Cardiotonic Pills on Chest Pain and Discomfort: A Multi-center Double-blind Randomized Controlled Trial [in Korean]," *Journal of Korean Oriental Internal Medicine*, vol. 26, no. 2, pp. 95-104, 2005.
6	J.H.P, M.J.L, H.J.K, et al, " Efficacy and Adverse Events of Bangpungtongseong-san(Bofutsusho-san) and Bangkihwangki-tang(Boiogiot-tang) by Oriental Obesity Pattern Identification on Obese Subjects : Randomized, Double Blind, Placebo-controlled trial [in Korean]," *Journal of Korean Oriental Medical Society*, vol.26, no.3, pp.249-262, 2005.
7	Y.C.P, " Effect of Socheongryong-tang on Punghan and Pungyeol Type Common Cold : A Double Blind, Placebo Controlled Study [in Korean]," *Korean Journal of Oriental Physiology & Pathology*, vol.19, no.2, pp.524-529, 2005.
8	M.J.P, J.W.S, I.H.S, et al, " "The Clinical Study on the Effect of Sulguanjul-bang No.1 According to Formulation [in Korean]," *The Korean Journal Of Oriental Medical Prescription*, vol.13, no.2, pp.193-206, 2005.

9 C.S.Y, L.C.H, J.J.Y, et al, " Effects of Ephedra on Weight Loss and Heart Rate Variability : A Double-Blind Randomized Controlled Pilot Study [in Korean]," *The Journal of Korean Oriental Internal Medicine*, vol.27, no,4, pp.836-844, 2006.

10 D.S.H, M.W.P, H.N.C, et al, " Effects of Mokhyangsaenghwa-tang geverage on Postpartum Recovery and Lactation [in Korean]," *The Journal of Oriental Obstetrics and Gynecology*, vol.19, no.4, pp.216-224, 2006.

11 D.H.S, L.C.H, L.H.K, et al, " Effect of Mahuang on Heart Rate Variability in Adults: a Double-Blind, Placebo-Controlled, Randomized Trial [in Korean]," *Journal of Korean Oriental Internal Medicine*, vol. 28, no. 1, pp. 105-116, 2007.

12 S.H.S, J.H.K, M.B.K, et al, " A Clinical Research about the Effects of Seunggaltang on Patients with Atopic Dermatitis [in Korean]," *The Journal of Korean Oriental Medical Ophthalmology &*

Otolaryngology & Dermatology vol.20, no.2, pp.199-212, 2007.

13 C.H.K, D.S.H, J.T.K, et al, " A Randomized Study, Double-Blind, Placebo-Controled Study to

Herbal Shampoo & Essence about Dandruff [in Korean]," *The Journal of Korean Oriental Medical Ophthalmology &Otolaryngology & Dermatology*, vol.20, no.3, pp.222-235,2007.

14 J.J.Y, L.C.H, C.S.Y, et al, " Effects of Mahuang fo Weight Loss in Healthy Adults : A Double-Blind, Controlled, Randomized, Clinical Trial [in Korean]," *Journal of Korean Oriental Medical Society*, vol.28, no.1, pp.63-71, 2007.

15 L.C.H, C.S.Y, T.H.L, et al, " Short-Term Effects of Mahuang on State-Trait Anxiety According to Sasang Constitution Classification : A Double-Blind Randomized Controlled Trial [in Korean]," *Journal of Korean Oriental Internal Medicine*, vol.28, no.1, pp.106-114, 2007.

16 C.S.Y, L.C.H, H.S.J, et al, " Effects of Ephedra on the State-Trait Anxiety in Female Adults : A Double-Blind Randomized Controlled Study [in Korean]," *Journal of Korean Oriental Internal Medicine*, vol.28, no.2, pp.346-353, 2007.

17 D.C.Y, H.T.K, E.H.K, et al, " Clinical Research of Atopic Dermatitis Treated by Hwangryeonhaedok-Tang in Cosmetics [in Korean]," *Korean Journal of Oriental Physiology & Pathology e*, vol. 22, no. 6, pp. 1611-1620, 2008.

Randomized Clinical Trials on Herbal Medicine in Korean Literature:

Bibliometric Analysis and Methodological Quality

**No**	**Included article**

18 S.H.K, D.C.Y, H.T.K, et al., " A Clinical Research of Atopic Dermatitis treated by Yeongyuseungmatang in cosmetics [in Korean]," *The Journal of Korean Oriental Medical Ophthalmology & Otolaryngology & Dermatology*, vol. 21, no. 2, pp. 126-141, 2008.

19 H.H.B, W.C.K, Y.C.P, et al, " Effectiveness of a Yeonkyopaedok-san Extract in the Treatment of The Common Cold : Results of a Community-based, Double Blind, Randomized Placebo Controlled Trial [in Korean]," *Korean Journal of Oriental Physiology & Pathology*, vol.22, no.1, pp.234-245, 2008.

20 S.B.H, S.M.P, M.J.O, et al, " A Double-blind, Randomized, Placebo-controlled Intervention Study of the Efficacy and Safety of 'Atomento solution & cream' in patients with Atopic Dermatitis [in English]," *The Journal of Korean Oriental Medical Ophthalmology & Otolaryngology & Dermatology*, vol. 22, no. 2, pp. 223-237, 2009.

21 T.H.L, L.C.H, C.S.Y, et al, " Adverse Effects of Ephedra According to SaasngTypologyin Healthy Adults : A Double-Blind Randomized Controlled Trial " [in Korean]," *Journal of Korean Oriental Internal Medicine*, vol.30, no.1, pp.144-152, 2009.

22 J.E.L, Y.K.S, H.H.L, et al, " Randomized, Double Blind, Placebo-Controlled Study of Bofu-tsusho-san on Obese Patients [in Korean]," *Journal of Society of Korean Medicine for Obesity Research*, vol.10, no 1, pp.1-16, 2010.

23 J.E.L, Y.K.S, H.H.L, et al, " Clinical Trial of Taeeumjowui-Tang(Taiyintiaowei-tang) on Obese Patients - Randomized, Double Blind, Placebo-Controlled Study [in Korean]," *Journal of Oriental Rehabilitation Medicine*, vol. 20, no. 2, pp. 145-160, 2010.

24 Y.M.K, Y.C.P, J.H.J, et al, " Effect of Herb Medicine Treatment for Functional Dyspepsia : A Randomized Placebo-Controlled and Compared Standard Treatment Trial [in Korean]," *Journal of Korean Oriental Medical Society*, vol.31, no.1, pp.01-13, 2010.

25 I.H.C, S.H.K, Y.C.K, et al, "The A Clinical Study of Shi Ho Cheong Gan-San on Blood Heat Pattern Atopic Dermatitis: a randomized, double-blind clinical trial [in English]," *The Journal of Korean Oriental Medical Ophthalmology & Otolaryngology & Dermatology*, vol.24, no.1, pp.96-110, 2011.

26 J.H.Park, C.Y.L, H.J.K, et al, " Impact of GNB3, ADRB3, UCP2, and PPARγ-Pro12Ala polymorphisms on Boiogito response in obese subjects : A randomized, double-blind, placebo-controlled trial [in Korean]," *Journal of Oriental Rehabilitation Medicine*, vol.21, no.2, pp.265-278, 2011.

27 D.H.L, H.J.H, L.H.K, et al, " Effect of Saengmaeg-san Extract on Xerostomia in Stroke Patients : A Double-Blind Randomized Controlled Study [in Korean]," *Journal of Korean Oriental Internal Medicine*, vol.32, no.4, pp.542-549, 2011.

28 J.S.L, E.J.D, M.A,K, et al, " Clinical Trial to Evaluate the Efficacy of Extract of Citri

Pericarpium on Serum Lipid Profiles in Subjects : a Randomized, Double-blind [in Korean]," *The Korea Association of Herbology*, vol.26, no.1, pp.125-132, 2011.

29 H.S.J, S.Y.K, H.J.H, et al, " The Effects of SBD-1 on Hangover Syndrome : a Randomized Double-blind Crossover Prelimninary Study [in Korean]," *Journal of Korean Oriental Internal Medicine*, vol.33, no.4, pp.511-519, 2012.

30 J.H. Park, S Boss, C.Y. Lim et al"Impact of GNB3, ADRB3, UCP2, and PPARγ-Pro12Ala polymorphisms on Boiogito response in obese subjects : A randomized, double-blind, placebo-controlled trial[in Korean]" *Journal of Society of Korean Medicine for Obesity Research*, vol.12, no. 2, 2012.

Randomized Clinical Trials on Moxibustion Therapy in Korean Literature:

Bibliometric Analysis and Methodological Quality (N=12)

No	Included article
1	K. S. Kang, E. J. Jeong, S. K. Moon et al, "Clinical Study on the Effects of Moxibustion for Post-stroke Voiding Dysfunction [in Korean]," *Journal of Korean Oriental Medical Society*, vol. 21, no. 4, pp. 236-241, 2000.
2	S. I. Kang, C. W. Lim, W. T. Park et al, "The Clinical study of acupuncture and moxibustion therapy on ch'ǒnch'u (ST25) for constipation [in Korean]," *The Journal of Korean Acupuncture and Moxibustion society*, vol. 18, no. 6, pp. 125-134, 2001.
3	Y. S. Kim, C. H. Kim, and K. S. Kim, "Effect of Auricular Acupuncture on Postoperative Nausea and Vomiting [in Korean]," *Korean Academy of Fundamentals of Nursing*, vol. 10, no. 2, pp. 244-253, 2003.
4	Y. S. Choi, T. K. Kim, W. S. Jung et al, "Effects of Moxibustion on the Hemiplegic Upper Extremity After Stroke [in Korean]," *Journal of Korean Oriental Internal Medicine*, vol. 24, no. 2, pp. 283-289, 2003.
5	B. S. Kim, I. S. Jang, J. J. Yeo et al, "Effect of Choksamni(足三里, ST36) Moxibustion on Blood Pressure Elevation in Hypertensive Patients: A Randomized Controlled Trial [in Korean]," *Journal of Korean Oriental Medical Society*, vol. 26 no. 3 pp. 66-

73,2005

6 S. H. Lee, J. K. Kim, Y. H. Son et al, "A Clinical Study of Moxibustion Therapy's Effect on Functional Recovery in Hemiplegia on Stroke [in Korean]," *Journal of Korean Oriental Internal Medicine*, vol. 29, no. 1, pp. 278-284, 2008.

7 J. H. Cho, H.S. Kim, D. Y. Choi, et al., "A Clinical Study on the Effect of Aroma Ceramic Moxibustion for Primary Dysmenorrhea [in Korean]," *The Journal of Oriental Obstetrics and Gynecology*, vol. 22, no. 1, pp. 19-27, 2009.

8 G. C. Kim, Y. S. Kim and H. W. Lee, "A Comparative Study on The Effect of Cupping and Moxibustion Therapy on Treating Constipation of Stroke Patients [in Korean]," *The Korean Society for Oriental Preventive Medicine*, vol. 13, no. 3, pp. 101-112, 2009.

9 Y. S. Kim, K. B. Kim, G. C. Kim et al, "Effects of the BUDDEUMI Therapy on the Relief of Shoulder Pain among Middle Aged Women [in Korean],"*Korean Journal of Oriental Physiology & Pathology*, vol.23 , no. 5, pp. 1172-177, 2009.

10 J. S. Park, S. N. Woo, H. J. Yeo et al, "The Effects of Hand Moxibustion Therapy on Knee Joint Pain Joint Range of Motion and Discomfort during ADL in Elderly People[in Korea]," *The Korean Society for Oriental Preventive Medicine,* vol. 13, no. 1, pp.81-92, 2009.

11 S. Y. Cho, J. Y. Jang, S. J. Kim et al, "Effect of PC6 Moxibustion for Mental Stress on Short-term Analysis of Heart Rate Variability [in Korean]," *The Journal of Korean Acupuncture and Moxibustion Society,* vol. 27, no. 2, pp. 51-58, 2010.

12 J. J. Lee, S. J. Kim, O. J. Park et al, "The Effect of Moxibustion at Jeonjung(CV17) on the Heart Rate Variability in Healthy Adults [in Korean]," *The Journal of Korean Acupuncture and Moxibustion society*, vol. 29, no. 4, pp. 43-53, 2012.

Appendix S5. RCTs included in bibliometric analysis and quality assessment

Randomized Clinical Trials on Cupping Therapy in Korean Literature:

Bibliometric Analysis and Methodological Quality (n=5)

No	Included article
1	J. K. Oh, S. S. Kim, "Effect of negative therapy at back meridian points on blood gas components and immune functions in male college students [in Korea]," *Journal of Korean Oriental Medical Society,* vol. 20, no. 1, pp.75-83, 1999.
2	J. A. Shin, Y. G. Lee, "Decreasing Effect of Wet-Cupping at Daechu(大椎, Dazhui:GV14)-point on the Increase of Blood Pressure in Patients with Stroke [in Korean]," *Journal of Korean Oriental Medical Society*, vol. 23, no. 3, pp. 85-95, 2002.
3	D. Y. Son, M. S. Kim, S. J. Kim et al, "Comparison of Depletion and Cupping Therapy with Cupping Therapy on Acute Lumbosacral Strain Patients [in Korean],"*Journal of Oriental Rehabilitation Medicine*, vol.13 , no. 4, pp. 53-61, 2003.
4	J. S. Hwang, D. J. Lim, J. H. Hwang et al, "Study of the Effect of Blood-letting Cupping Treatment for Acute Ankle Sprain: A Randomize Controlled Trial [in Korean]," *The Journal of Korean Acupuncture and Moxibustion society*, vol. 22 no. 3 pp. 243-252,2005
5	E. J. Lee, T. D. Jeoun, I. J. Yoon et al, "The Clinical Effects of Cupping Therapy at Bedtime for Sleep Disorder Induced by Traffic Accidents [in Korean]," *The Journal of Korean Acupuncture and Moxibustion society*, vol. 20, no. 1, pp. 193-208, 2010.

No	Randomized Clinical Trials on Chuna Therapy in Korean Literature: Bibliometric Analysis and Methodological Quality (N=17)<br>Included article
1	W. J. Sim, S. H. Moon, H. J. Ryu et al, "The Effects of the Muscles along Meridians Release Therapy to Spastic Patients after Stroke [in Korea]," *Journal of Oriental Rehabilitation Medicine,* vol. 14, no. 1, pp.63-75, 2004.
2	D. Y. Lee, S. J. Kim, "Clinical Study on the Ankle Joint Traction Therapy for Ankle Sprain [in Korean]," *Journal of Korean Oriental Medical Society*, vol. 15, no. 4, pp. 39-46, 2005.
3	T. S. Kim, C. Y. Kim, K. H. Lee et al, "Comparative Clinical Study between the Acupuncture Treatment and the Chuna Treatment on Temporomandibular Disorder [in Korean]," *The Journal of Korea Chuna Manual Medicine for Spine and Nerves*, vol. 1 no. 1 pp. 55-64,2006
4	H. S. Ryu, S. H. Jeon, D. S. Park et al, "Clinical Study for Chuna Treatment on Neck Pain Patient with Hypolordotic Cervical Spine [in Korean],"*The Journal of Korea Chuna Manual Medicine for Spine and Nerves*, vol.1 , no. 2, pp. 11-20, 2006.
5	O. G Kwon, M. S. Kim, H. D. Ahn et al, "The Efficacy of Sacro-Occipital Technique for Stroke Patients on Activity of Daily Living(ADL), Leg Function Balance [in Korean]," *The Journal of Korea Chuna Manual Medicine for Spine and Nerves*, vol. 2, no. 2, pp.

57-68, 2007.

6 M. B. Kim, S. H. Chung, S. S Kim, "The Influences of Chuna(shoulder traction) Therapy for Shoulder Pain and Range of Movement in Hemiplegic Patients After Stoke [in Korean]," *Journal of Oriental Rehabilitation Medicine*, vol. 17, no. 2, pp. 185-198, 2007.

7 J. M. Lee, J. H. Park, E. J. Lee et al, "Effect of Chuna Treatment(Manipulation) on Lumbar Sprain caused by Traffic Accident in Early Stage [in Korean]," *The Journal of Korea Chuna Manual Medicine for Spine and Nerves*, vol. 4, no. 2, pp. 61-74, 2009.

8 O. G. Kwon, W. S. Jang, C. H. Woo et al., "The Efficacy of Adjusting Leg Length Inequality by Chuna Manual Treatment for Post-Stroke Hemiplegia [in Korean]," *Journal of Oriental Rehabilitation Medicine*, vol. 19, no. 2, pp. 187-202, 2009.

9 D. Y. Yoon, J. M. Son, J. S. Choi et al, "Effect of Chuna Treatment(Manupulation) on Lumbar Sprain Caused by Traffic Accident [in Korean]," *The Journal of Korea Chuna Manual Medicine for Spine and Nerves*, vol. 5, no. 1, pp. 23-30, 2010.

10 J. H. Woo, K. W. Han, E. S. Kim et al, "A Clinical Study Comparing the Effects of Chuna Treatment and Ouhyul Herbal Acupuncture on the Patients with Cervical Pain Caused by Traffic Accidents [in Korean]," *The Journal of Korean Acupuncture and Moxibustion Society*, vol. 28, no. 5, pp. 9-18, 2011.

11 E. S. Kim, T. H. Kim, S. C. Lee et al, "Effect of Chuna Manupulation on Cervical Sprain Caused by Traffic Accident [in Korean]," *Society of Sports Korean Medicine*, vol. 11, no. 1, pp.49-56, 2011.

12 E. S. Jin, J. H. Lee, H. K. Kim et al, "The Clinical Study for the Effects of Chuna Treatment on Patients with Temporomandibular Disorder [in Korean]," *Journal of Oriental Rehabilitation Medicine*, vol. 21, no. 1, pp. 125-135, 2011.

13 J. S. Kim, J. H. Lee, K. Y. Yang et al, "The Comparative Study on the Effect of Pharmacopuncture Treatment and Chuna Treatment for Low Back Pain caused by Traffic accidents [in Korean]," *The Journal of Korea Chuna Manual Medicine for Spine and Nerves*, vol. 6, no. 2. pp. 155-164, 2011.

14 D. H. Chang, U. Y. Bae, J. H. Jung et al, "The Effects of Burning Acupuncture Therapy with Chuna Therapy for Low Back Pain Patients [in Korean]," *Journal of Oriental Rehabilitation Medicine*, vol. 21, no. 3, pp. 21-32, 2011.

15 J. H. Lee, J. S. Kim, K. Y. Yang et al., "The Comparative Study on the Effect of Pharmacopuncture Treatment and Chuna Treatment for Neck Pain Caused by Traffic Accidents[in Korean]," *Journal of Oriental Rehabilitation Medicine,* vol. 22, no. 1, pp. 75-83, 2012.

16 S. H. Yun, W. S. Jung, S. H. Cho et al, "The Clinical Study for Chuna Treatment on

	Patients with Cervicogenic Headache Caused by Traffic Accident [in Korean]," *Journal of Oriental Rehabilitation Medicine*, vol. 22, no. 2, pp. 165-175, 2012.
17	J. S. Lee, S. J. Lee, J. H. Nam et al, "The Comparative Study on the Effect of Pharmacopuncture Treatment, Chuna Treatment, Pharmacopuncture – Chuna Cooperative Treatment for Neck Pain Caused by Traffic Accidents [in Korean]," *The Journal of Korean Acupuncture and Moxibustion society*, vol. 29, no. 5, pp. 87-95, 2012.

Randomized Clinical Trials on Massage Therapy in Korean Literature:

Bibliometric Analysis and Methodological Quality (N=10)

No	Included article
1	B. C. Yoon, J. H. Lee, Y. W. Ham et al, "Effects of massage and microwave diathermy therapy on eccentric exercise-induced DOMS and indices of muscle damage [in Korea]," *The Korean Academy of Physical Therapy Science,* vol. 13, no. 2, pp.293-303, 2001.
2	K. J. Sohn, Y. S. Choi, M. J. Kim et al, "The Effects of Aroma Self Massage in Hands on Pain, Depressive Mood and Anxiety in Breast Cancer Patients [in Korean]," *Journal of Hospice and Palliative Care*, pp. 18-29, 2005
3	S. J. Kim, Y. D Park, "The Effects of Swedish Massage on the Changes of Autonomic Nervous System in the Athletic Speciality Group Students [in Korean]," *Korea Coaching Development Research*, vol. 9, no. 3, pp. 201-208, 2007.
4	M. S. Song, N. C. Kim, "Effect of Hand Massage on Fatigue, Sleep Satisfaction and Blood Pressure of the aged in a Long-term Care Facility [in Korean]," *Korean Journal of Adult Nursing*, vol. 21 no. 2 pp. 179-186,2009
5	M. B. Kim, K. K. Kim, S. H. Hong et al, "The Clinical Tests for Treatment Improvement Comparison in Myopia between Eye Acupuncture Massage Machine(NURIEYE-1) and Acupuncture [in Korean],"*The Journal of Korea Oriental Medical Ophthalmology & Otolaryngology & Dermatology*, vol. 22 , no. 3, pp. 80-94, 2009.
6	I. S Jung, J. S. Kim, H. S. Yoo, "Effect of Auricular Acupress Therapy on Insomnia of Cancer Patients : Randomized, Single Blinded, Placebo Controlled Trial [in Korean]," *Journal of Pharmacopuncture*, vol. 13, no. 2, pp. 93-100, 2010.
7	W. A. Kwon, D. D. Kim, J. H. Lee, "The Effects of Massage and Satic Stretching on Cervical Range of Motion in Their 20s of Normal Adult [in Korean]," *Journal of the*

	Korea Academia-Industrial cooperation Society, vol. 11, no. 11, pp. 4346-4353, 2010.
8	M. Y. Chung, E. S. Choi, "A Comparison between Effects of Aroma Massage and Meridian Massage on Constipation and Stress in Women College Students [in Korean]," *Journal of Korean Academy of Nursing*, vol. 41, no. 1, pp. 26-35, 2011.
9	M. Y. Chung, "Effects of Aroma Massage on Constipation in Women College Students [in Korean]," *Journal of Korean Academy of Rural Health Nursing*, vol. 6, no. 2, pp. 78-86, 2011.
10	D. W. Lee, J. H. Park, S. N. Eom et al, "Effect of Combined Stimulus on Stress Relief [in Korean]," *Journal of Biomedical Engineering Research*, vol. 33, pp. 194-201, 2012.

Randomized Clinical Trials on Taping Therapy in Korean Literature:

Bibliometric Analysis and Methodological Quality (N=7)

**No**	**Included article**
1	Y. H. Park, H. D. Shin, S. S. Kim et al, “Antispastic Effect of Taping on Spasticity in Stroke Patients [in Korea],” *Journal of Oriental Rehabilitation Medicine,* vol. 11, no. 1, pp.159-171, 2001.
2	K. S. Park, E. N. Ryoo, M. H. Choi, “The Effect of Balance Taping Therapy on Pain of the Lower Back Pain Patient [in Korean]," 2001.
3	J. W. Lee, C. H. Kim, S. H. Moon et al, "Effectiveness of Spiral Taping in the Low Back Pain Patients [in Korean],"*The Journal of Korean Acupuncture & Moxibustion Society*,

vol. 23 , no. 5, pp. 1-10, 2006.

4 J. M. Park, M. S. Yoon, I. Woo et al, "The Effect of Ankle Meridian Tendino-musculature Taping on Lateral Ankle Sprain; Randomized Controlled clinical study [in Korean],"*Journal of Oriental Rehabilitation Medicine*, vol. 16 no. 1 pp. 63-71,2006.

5 M. C. Park, E. Y. Kim and M. S. Ha, "Effects of Taping Therapy by Methos on Back Pain and Muscle Flexibility of Bus Drivers's [in Korean]," *Journal of the Korea Academia-Industrial cooperation Society*, vol. 11, no. 11, pp. 4346-4353, 2010.

6 D. H. Kwon, S. M. Lee, H. J. Kim et al, "Effect of Microcurrent Taping Therapy on Lower Back Pain [in Korean]," *Journal of Korean Oriental Medical Society*, vol. 32, no. 5, pp. 114-125, 2011.

7 M. Ko, J. Lee, "Effect of Balance Taping Therapy on Neck Pain in High School Students [in Korean]," *Journal of Korean Academy of Adult Nursing*, vol. 24, no. 4, pp. 417-427, 2011.

No	Included article
	**Randomized Clinical Trials on Gi-gong Therapy in Korean Literature: Bibliometric Analysis and Methodological Quality(N=28)**
1	S. J. Kang, S. Y. Song, B. C. Sim, "The Effect of Special Breathing Method (Dangeon)and Chinese Medicine Ingestion on Athletic Performance [in Korean],"*Korea national university researh center*, vol. 5, no. 1, pp. 1-21, 1986.
2	K. Y. Kim, "Effects of Danjeon Breath on the Basic Physical Strength Improvement [in Korean]," *Myongji university collection of dissertation*, 1986.
3	H. Choi, " The influence of meditation and Gukseondo on middle school student's concept of ego, anxiety, aggression [in Korean]," *Chonam national university pedagogy science collection of dissertation*, 1992.
4	S. C. Lee, " The influence of meditation training program on weightlifting record and Physiological Arousal [in Korean]," *Hanyang university sports science collection of dissertation*, 1993.
5	Y. G. Choi, N. S. Pyo, "The Effects of Kuk Sun Do Don-jeon Breathing Training on Anxiety and Heart Rate [in Korean]," *Journal of Physical Education & Sports Science*, vol. 10, pp. 173-184, 1994.
6	Y. G. Choi, " The Effect of Danjeon Breathe to anxiety, blood pressure, EEG, blood lipid metabolism [in Korean]," *Journal of Physical Education & Sports Science*, 1995.
7	C. H. Jo, N. S. Pyo and C. Y. Park, "The Effects of Dan-Jeon Breathing Training in Performing on the Self-Paced Tasks [in Korean],"*Journal of Physical Education & Sports Science*, vol. 13, pp. 131-140, 1997.
8	Y. H. Kim, "The Effect of Danjeon Breathe and Qi Dance to the Juvenile Character Formation [in Korean]," *The Journal of Korean Society of Jungshin Science*, vol. 9, pp. 99-116, 1998.
9	C. H. Cho, J. S. Park, "The Effect of Dan-jun Breathing on the Cardiorespiratory Function [in Korean],"*Journal of Sport and Leisure Studies*, vol. 12, no. 1, pp.731-743, 1999.

10 J. H. Park, K. H. Lee, "The Effects of Taichi Training on Self – concept in Elementary School Boys [in Korean]," *Korean Society of Sport Psychology*, vol. 11, no. 1, pp. 155-169, 2000.

11 J. S. Park, C. H. Cho, "Effects of Dan-jun Breathing Training and Sport Activities on the Youngsters' Mental Health [in Korean]," *Journal of Sport and Leisure Studies,* vol. 14, no. 1, pp. 155-165, 2000.

12 K. H. Lee, K. W. Kim, Y. J. Choi, "Dan Jeon Breathing Technique to Reduce Perimenstrual Discomfort : Impact on nursing intervention [in Korea]," *The Korean Academy of Physical Therapy Science,* vol. 12, no. 1, pp.17-32, 2000.

13 J.S. Park, C. H. Cho, " The Effect of Danjeon Breathe to National Archer' Concentration Strategies [in Korean]," *Korea Sports Development*, vol. 11, no. 3, pp. 75-86, 2000.

14 S. Y. Cha, C. H. Cho, "The effect of Dan-jeon breathing training on 1000m run performance in middle school man [in Korean]," *Journal of Sport and Leisure Studies,* vol. 14, no.1, pp. 691-698, 2000.

15 J. W. Park, H. Y. Cho, "A study on the Digital Infrared Thermal Image in Korean Healthy Men treated with several cupping therapy [in Korean]," *Journal of Korean medical Gi-gong Academy*, vol. 5, pp. 1-21, 2001.

16 J.Y. Shim, "A study on the effect of brain respiration training on respiratory circulation function", *Korea sport resarch*, vol. 12, No. 4, 2001.

17 Y.S. Jin, J.Y. Park, H.Y. Kim et al, "The Effect of Aerobic Exercise and Qi-Gong on Lymphocyte, β-adrenergic Receptor, Antioxidative Function, and Heart Rate Variablility in the Elderly", *The Korean Journal of Sports Medicine*, vol. 19, no.2 , 2001.

18 C.H. Cho, J.S. Park, "Effects of Dan-Jeon Breathing Training on Dart-throwing Performance [in Korean]", *Korea sport research,* vol. 12, no. 4. pp99-108, 2001

19 C.H. Cho , J. S. Park et al, "Effects of Dan-jeon Breathing and Sensory Image Training on Motor Learning in Self-paced Tasks[in Korean]", *Korean society of sport and leisure studies,* vol.18, no. 2 , pp1393-1403, 2002.

20 H. S. Jang, M. S. Lee, J. W. Jang et al, "Acute effects of Qi-therapy on psychological symptoms and pain in the older subjects[in Korean]", *Journal ot the Korean society of jungshin scienc,* vol. 6, no. 2, pp 15-20, 2002.

21 J.Y. Shim , "The effects of brain respiration training on immune cells and stress hormone[in Korean]", *Jusun university research* , 2002.

22 S. J. Kim, Y. D Park, "The Effects of Swedish Massage on the Changes of Autonomic Nervous System in the Athletic Speciality Group Students [in Korean]," *Journal of the*

	Korean Society of Jungshin Science, vol. 6, no. 2, pp.15-20, 2002
23	E. Y. Jung, S. S. Na and K. N. Lee, "Clinical effect of Gigong therapy by measuring ABR-2000 on Neck Stiffness Patients [in Korean],"*The Journal of Korean Academy of Medical Gi-gong*, vol. 7 , no. 1, pp. 61-76, 2003.
24	M. N. Kim, "The effect of Qi prenatal education – Qi Tae Gyo : Qing Jing Gong – on the physical & psychological conditions of pregnant women [in Korean]," *The Journal of Korean Academy of Medical Gi-gong*, vol. 7 no. 2 pp. 111-133,2004
25	J.Y. Shim, "Effects of Dhanhak Qigong Training on Cognition of Daily Stress Inventory(DSI) and Heart Rate[in Korean]" , *Korea sport research* , vol. 15, no. 2,pp1229-1239, 2004.
26	J.Y. Shim , "The Effects of Brain Development Program on stress of elderly office men [in Korean]", *Korea sport research* , vol. 14, no. 4,pp1341-1354, 2004.
27	J.Y. Shim , "*Analysis* of Stress Hormone Reaction According to DoIn Exercise and *Meditation* Program [in Korean]", *Korea sport research* , vol. 14, no. 6,pp1463-1473, 2004.
28	S. Y. Cha, C. H. Cho, "The effects of the Dan-jeon breathing training condition on golf-putting performance[in Korean]," *Journal of Oriental Rehabilitation Medicine*, vol. 27, no. 2, pp. 527-539, 2011.

Randomized Clinical Trials on Magnetic field in Korean Literature:

Bibliometric Analysis and Methodological Quality (N=3)

**No**	**Included article**
1	H.K.K, E.S.L, " The Effects of the Magnetic Therapy Treated on ear acupuncture points for exercise pre,post fatigue substance [in Korean]," *Journal of Oriental Rehabilitation Medicine*, vol.8, no.1, pp.187-202, 1998.
2	S.R.C, E.S.L, "The Effects of the Magnetic Therapy Treated on Acupuncture Points for Recovery from Fatigue with before and after Exercise [in Korea]," *Journal of Oriental Rehabilitation Medicine*, vol.8, no.1, pp.158-170, 1998.
3	K.H.H, H.J.H, Y.H.P, et al, " Effects of Pulsed Electromagnetic Therapy for Cervicogenic Headaches : Randomised Clinical Trial [in Korean]," *Journal of Oriental Rehabilitation Medicine*, vol.17, no.3, pp.147-159, 2007.

Randomized Clinical Trials on Meditation Therapy in Korean Literature: Bibliometric Analysis and Methodological Quality (N=2)	
No	**Included article**
1	S.J.H, W.T.S, M.S.O et al, "The Effect of Yukjageul Respiration on the Difference of Skin Temperature [in Korean]," *Journal of Oriental Rehabilitation Medicine*, vol.11, no.1, pp.53-71, 2001.
2	H.N.P, S.J.Y, "Effects of Meditation Training Program on Self Concept, Abstinence Self-efficacy, and Abstinenece in Alcoholic Patients [in Korea]," *Journal Korean Academy Psychology Mental Health Nusrsing*, vol.14, no.3, pp.304-312, 2005.

Randomized Clinical Trials on Yoga Therapy in Korean Literature: Bibliometric Analysis and Methodological Quality (N=3)	
No	**Included article**
1	H.J.K, "Effect of YogaExerciseprogram on Dysmenorrhea,Menstrual Pain and Vasopressin oftheFemaleUniversity Students [in Korean]," 淑明女大 教育論義, vol.20, 2009.

2	E.M.K, C.H.C, "Effects of Yogic Breathing on Stress: in Relation to EEG Quotients [in Korea]," *Korean Society for Emotion&Sensibility*, Spring Conference, Poster Session 1, pp.85-86, 2012.
3	H.S.S, J.Y.K, "The Effects of Yoga Exercise on Balance and Gait Velocity in Stroke Patient [in Korea]," *Korean Journal of the Korea Academia-Industrial cooperation Society*, vol.14, no.1, pp.294-300, 2013.

Randomized Clinical Trials on Tai-chi in Korean Literature:

Bibliometric Analysis and Methodological Quality (N=1)

No	Included article
1	E.N.L, "The Effect of Tai Chi Exercise Program on Blood Pressure, Total Cholesterol and Cortisol Level in Patients with Essential Hypertension [in Korean]," *Korean Academy of Fundamentals of Nursing*, vol.34, no.5, pp.829-837, 2004.

Randomized Clinical Trials on Aroma Therapy in Korean Literature:

Bibliometric Analysis and Methodological Quality (N=4)

No	Included article
1	M.A.K, M.S.K, B.S.K, et al. "Effect of Lavender Essential Oil Autonomic Nervous System by Power Spectrum Analysis of Heart Rate Variablility in Healthy Adults : A Randomized Controlled Trial, [in Korea]," *Journal of Korean Oriental Internal Medicine*, vol.24, no.3, pp.569-578, 2003.
2	H.J.K, "Effect of Aromatherapy Massage on Abdominal Fat and Body Image in Post-menopausal Women [in Korea]," *Korean Academy of Fundamentals of Nursing*, vol.37, no.4, pp.603-612, 2007.
3	E.H.C, "Comparison of Effects Lavender Abdominal Massage and inhalation on Dysmenorrhea, Pain, Anxiety and Depression [in Korean]," *Korean Academy of Fundamentals of Nursing*, vol.16, no.3, pp.300-306, 2009.
4	J.H.C, M.J.K, H.S.K, et al. "Effects of Aromatherapy in blending oil of Basil, Lavender, Rosemary, and Rose on Headache, Anxiety and Serum Cortisol level in the Middle-

Aged Women [in Korea]," *Korean Society of Biological Nursing Science*, vol.12,no.3, pp.133-139, 2010.

Randomized Clinical Trials on Ginseng therapy in Korean Literature:

Bibliometric Analysis and Methodological Quality (N=19)

No	**Included article**
1	J.C. Seo, S.W Han, J.S. Byun et al, "Double-blind randomized controlled trials on superficial body temperature of Korean ginseng and American ginseng in Koreans and chineses[in Korean]", *The Korea Association of Herbology*, vol. 18, No 2. Pp71-86, 2003.
2	J. C. Seo, Z.J. Heo, S.W. Han et al, "The effect of Korean ginseng and American ginseng on body temperature in Korean and chinese-Double blind randomized controlled trials-[in Korean]", *Korean Journal of Oriental Physiology & Pathology*, vol.17, No. 3, pp837-841, 2003.
3	J. C. Seo, S. Y. Yee, K.M Lee et al, "Double-blind randomized controlled trials on blood pressure and pulse rate of Korean ginseng and American ginseng in Korean and chinese[in Korean]", *Korean Journal of Oriental Physiology & Pathology*, vol.18, No. 2, pp606-611, 2004.
4	J. C. Seo, S. W. Han, J.S Byun, "The effect of ginseng and American ginseng on general symptom in Koreans and Chineses-Double blind Randomized controlled trials[in Korean] ", *Jounal of ginseng research*, vol. 29, No. 1, pp27-36, 2005.
5	K.H. Cho, W. S. Jung, Y.S. Kim, "The effect of ginseng and American ginseng on high blood presure -Double blind Randomized controlled trials"[in Korean], "*korea jinseng research*", 2005
6	S.G.. Lee, Y. H. Cho, T.G Kang et al, "Analysis of Plasma Proteome before and after Oral Administration of Acidic Polysacharide from Panax Ginseng, Double-blind, Placebo-controlled, Randomised Trial[in English]", *Journal of Ginseng Research*, vol. 21, No. 5, pp1185-1193, 2007.

7 K.S. Shin, J.J. Lee, Y.R. Jin et al, "Effect of Korean Red Ginseng extract on blood circulation in healthy volunteers: A randomized, double-blind, placebo-controlled trial[in Korean]", *Jounal of Ginseng Research*, vol. 31, No. 2, pp10-116, 2007.

8 D. K. Kim, J A Lee, Y. B. Kim, "A randomized controlled trial assessing Korea red ginseng treatment of Helicobacter pylori-associated chronic gastritis[in Korean]", *The Korean Association of Internal Medicine*, vol 72, No 1, 2007.

9 H.S. Kim, Y.J. Yoon, J.M. Lee et al, "A clinical study on the effect of red ginseng for postmenopausal hot flushes[in English]", *J Orient Obstet Gynecol*, vol. 22, pp132-139, 2009.

10 W.S. Ham, W. T. Kim, J. S. Lee et al, "Efficacy and Safety of Red Ginseng Extract Powder in

Patients with Erectile Dysfunction: Multicenter, Randomized, Double-Blind, Placebo-Controlled Study[in Korean]", *Korean Journal of Urology*, vol. 50, No 2, pp159-164, 2009.

11 O.H. KJ, C. MJ, Lee HS, Hong HD et al, "Effects of Korean red ginseng on sexual arousal in menopausal women: placebo-controlled, double-blind crossover clinical study"[in English], "*J Sex Med*" vol. 7, No.4, pp1469-1477, 2010.

12 N.R. Kim, J. H. Kim, C. Y. Kim et al, "Effect of Korean Red Ginseng Supplementation on Ocular Blood Flow in Patients with Glaucoma[in English]", *Journal of Ginseng Research*, vol.34, No.3, pp237-245, 2010.

13 I.M. Chung, J. W. Lim, W. B. Pyun et al, "Korean Red Ginseng Improves Vascular Stiffness in Patients with Coronary Artery Disease[in English]", *Journal of Ginseng Research*, vol. 34, No3,pp212-218, 2010.

14 S.A. Lee, S. G. Kang, H. J. Lee, "Effect of Korean Red Ginseng on Sleep : A Randomized, Placebo-Controlled Trial[in Korean]", *Sleep Medicine and Psychophysiology*, vol 17, No 2, pp85-90, 2010.

15 J.H. Heo, S. T. Lee, M. J. Oh et al, "Improvement of Cognitive Deficit in Alzheimer's Disease Patients by Long Term Treatment with Korean Red Ginseng[in English]", *Journal of Ginseng Research*, vol. 35, No. 4, pp457-461, 2011.

16 D. H. Kwon, M.J. Lee, C. Y. Lim, "Efficacy of Red Ginseng by Oriental Medical Obesity Syndrome Differentiation on Obese women : Randomized, Double-blind, Placebo-controlled Trial[in Korean]", *Journal of Society of Korean Medicine for Obesity Research*, vol 11, No 1, pp01-14, 2011.

17 H.B. Yeo1, H. K. Yoon, H.J. Lee et al, "Effects of Korean Red Ginseng on Cognitive and Motor Function: A Double-blind, Randomized, Placebo-controlled Trial[in

English]”, *Journal of Ginseng Research*, vol.36, No.2, pp190-197, 2012.

Randomized Clinical Trials on Ginseng therapy in Korean Literature:

Bibliometric Analysis and Methodological Quality

No	**Included article**
18	D.H. Kwon, S. Boss , Mi. Y. Song et al, “Effi cacy of Korean Red Ginseng by Single Nucleotide Polymorphism in Obese Women: Randomized, Double-blind, Placebo-controlled Trial[in English]”, *Journal of Ginseng Research*, vol. 36, No. 2, pp176-189, 2012
19	S.Y. Kim , S.K Seo, Y.M. Choi et al, “Effects of red ginseng supplementation on menopausal symptoms and cardiovascular risk factors in postmenopausal women: a double-blind randomized controlled trial[in English]”, *Menopause*, vol. 19, pp461-466, 2012.

Randomized Clinical Trials on Vitamin& diet-based therapy in Korean Literature:

Bibliometric Analysis and Methodological Quality (N=5)

No	**Included article**
1	Y. K. Song, H. H. Lim, “The effect of diet and exercise training on body composition in obese women[in Korean]”, *Journal of Oriental Rehabilitation Medicine*, vol. 10, No.1, pp57-68, 2000.
2	Y. H. Baek, J. R. Kwak, S. J. Kim et al, “Effects of Kimchi supplementation and/or exercise training on body composition and plasma lipids in obese middle school girls[in Korean]”, *The Korean Society of Food Science and Nutrition*, vol. 30, No. 5, pp906-912, 2001
3	C.S. Kim, H.J. Kang, S.H. Lee et al, “The effect of Alpha-tocopherol supplementation on the improvement of antioxidant status and lymphocyte DNA damage in

postmenopausal women[in Korean]", *Journal of Korean Oriental Medical Society*, vol. 40, No. 8, pp708-718, 2007

4 M.Y. Chang , S.K. Park, T.J. Kwak et al, "Effects of the Mixture of Vitamin C, Vitamin E, Pycnogenol and Evening Primrose Oil on the UV-Induced Pigmentation and Wrinkle Reductions in Human Skin[in Korean]", *Korean Jounal of Nutrition*, vol. 42, No. 6, pp516-522, 2009.

5 H.M. Zeng, D.H. Kim, S.H. Lee et al, "Effects of Vanadium Water on Cancer-related Fatigue of Non Advanced Thyroid Cancer Patients: Randomized, Three armed,Triple Blinded Controlled Trial[in Korean]", *Daejeon university Korean medicine research*, vol. 20, No. 2, 2012.
